# Cytokeratin expression and distribution pattern of epithelioid macrophages in animals with different pathological forms of bovine paratuberculosis: potential role in resilience to PTB

**DOI:** 10.3389/fvets.2025.1690841

**Published:** 2025-12-11

**Authors:** Alejandra Isabel Navarro León, Marta Muñoz, Cristina Blanco-Vázquez, Natalia Iglesias, Tania Iglesias, María Canive, Gerard Badia-Bringué, Marta Alonso-Hearn, Ana Balseiro, Rosa Casais

**Affiliations:** 1 Centro de Biotecnología Animal, Servicio Regional de Investigación y Desarrollo Agroalimentario (SERIDA), Gijón, Asturias, Spain; 2 Unidad de Consultoría Estadística, Servicios científico-técnicos, Universidad de Oviedo, Gijón, Asturias, Spain; 3 Department of Animal Health, NEIKER, Basque Institute for Agricultural Research and Development, Basque Research and Technology Alliance (BRTA), Derio, Bizkaia, Spain; 4 Departamento de Sanidad Animal, Facultad de Veterinaria, Universidad de León, León, Spain; 5 Instituto de Ganadería de Montaña (IGM, CSIC-ULE), Finca Marzanas, León, Spain

**Keywords:** Paratuberculosis, cytokeratin, epithelioid macrophages, granuloma, resilience

## Abstract

Paratuberculosis (PTB) is a chronic enteritis caused by *Mycobacterium avium* subsp. *paratuberculosis* (Map). Genome-wide association studies revealed a significant enrichment of the keratinization pathway in cattle with multifocal lesions, suggesting a potential role of cytokeratins (CKs) in PTB resilience mechanisms. To confirm this, the amount of CK-expressing cells was analyzed in granulomas from the distal jejunum (DJE) and jejunal lymph nodes (DJELN) of animals with focal, multifocal, and diffuse lesions, and in control animals without lesions. Quantitative double-immunohistochemical (D-IHC) analysis [using Iba1 (ionized calcium-binding adapter molecule-1) and CK, as specific markers of macrophages and epithelial cells, respectively] showed that animals with multifocal lesions had the highest numbers of double-Iba1/CK positive cells [epithelioid macrophages (EMs)]. Significant differences were observed with the focal (*p* < 0.001), diffuse (*p* = 0.038), and control groups (*p* < 0.001) in JELN. Similarly, these animals showed higher numbers of single-CK expressing cells in JELN and DJE. Two EM distribution patterns were observed. In Pattern 1, mostly observed in animals with multifocal lesions (low Map load, no clinical signs), EMs form a barrier-like arrangement around the granuloma, while in Pattern 2, mainly found in animals with diffuse lesions (high Map load, clinical signs), EMs have a diffuse arrangement throughout the granuloma. These findings suggest that animals with multifocal lesions might represent a resilient phenotype that controls Map infection and disease progression. This is achieved through the formation of ordered granulomas that prevent Map dissemination and maintain tissue integrity, which are characteristic features of resilient animals. CK could be considered a potential biomarker of PTB resilience.

## 1 Introduction

Bovine paratuberculosis (PTB) is a chronic granulomatous enteritis caused by *Mycobacterium avium* subsp. *paratuberculosis* (Map). PTB has direct effects on animal health, causing significant economic losses in the dairy and livestock industries. PTB has potential public health implications since Map has been associated with several inflammatory and autoimmune diseases (1–4).

PTB, characterized by a chronic and granulomatous inflammation, progresses through four stages: silent, subclinical, clinical, and advanced clinical (5–7). Granulomas are well-organized, host-derived immune structures, primarily formed by macrophages, that vary in differentiation and phenotype, and may coexist with other immune cell types (8–10). Map-infected cattle show three main types of granulomatous lesions of increasing severity (focal, multifocal, and diffuse) according to González et al. (11), with diffuse lesions further subdivided into diffuse lymphoplasmacytic paucibacillary, diffuse intermediate, and diffuse histiocytic multibacillary lesions (12).

Macrophages are phagocytic cells that play a central role in the host’s defense, as part of the host’s innate immune response to Map infection (13). Map can hide within infected macrophages during the long subclinical stage of PTB (8, 13–15) with only a small percentage of exposed cattle developing clinical disease (14, 16). Jenvey et al. quantified the abundance and different phenotypes of macrophages in frozen bovine mid-ileum tissue sections of non-infected healthy cows, subclinically and clinically infected cows (17, 18). These authors showed that as the infection progresses, the number of macrophages significantly increases, but they fail to clear Map, which spreads from cell to cell, leading to further progression of the infection and the development of clinical disease.

PTB progression results in various changes of the macrophages within the granuloma (18), resulting in a range of histological appearances (11, 19). One major change is the ‘epithelioid’ transformation of macrophages into epithelioid macrophages (EMs), or epithelioid cells, characterized by flattened shape, ovoid nuclei, and membranes that interdigitate with adjacent cells. Although these cells are macrophage-derived, EMs are regarded as a specialized type of mononuclear phagocyte immobilized in the granuloma, with their function changing from phagocytosis to extracellular secretion (9). EMs express at least one canonical epithelial marker such as E-cadherin and cytokeratin (CK). The ability to express such protein markers is due to a process of macrophage polarization whereby macrophages change to a specific phenotype. This change results from a functional response to the microenvironmental stimuli and signals they encounter in each specific tissue (15), specifically through stimuli from a mycobacterial infection (20). For instance, E-cadherin is expressed in zebrafish macrophages during granuloma formation due to *Mycobacterium marinum* infection (21) and in skin granuloma macrophages of human specimens of sarcoidosis and foreign body granulomas. E-cadherin promotes homotypic aggregation of macrophages and multinucleated giant cells formation, crucial processes for disease pathogenesis (22). CK, a cytoskeletal protein highly specific to epithelial cells (23), has been previously used as a marker of EMs to study the expression of cellular components during chronic granulomatous inflammation in a teleost fish model (pacus, *Piaractus mesopatamicus*) induced by Bacillus Calmette-Guerin (BCG). This study demonstrated that macrophages with phagocytic activity can transform into epithelioid cells with secretory activity during infection (24). CK has also been used as an epithelioid cell marker in the ulcerative mycosis granulomas of Atlantic menhaden (25) and as a diagnostic marker of circulating tumor cells (26–28).

Genome-wide association studies revealed a significant enrichment of the keratinization pathway in cattle with multifocal lesions (29). Keratins (KRTs) belong to the largest subgroup of intermediate filament (IF) family of cytoskeletal proteins and represent the most abundant proteins in epithelial cells. They play a major role in maintaining gastrointestinal epithelial integrity, repairing tissue, and protecting cells from death. KRTs are also involved in stress responses and adaptation, as conveyed by severe disorders caused by inherited mutations in KRT genes (30). For instance, KRT5 expression in distal airway stem cells is essential for lung regeneration after H1N1 influenza virus infection (31), KRT7 may play a role in the histogenesis of small intestinal carcinoma associated with Crohn’s disease (32), and KRT75 has been associated with heat stress adaptation in Chinese cattle (33). Thus, CK expression could help contain the effects of Map infection. In this sense, infected animals have developed different resilience strategies to mitigate the negative impact of infections on host fitness: avoidance, resistance, and tolerance (34–38). Canive et al. (29) suggested that the CK overexpression in animals with multifocal lesions could provide tissue resilience by supporting the formation of “epithelioid granulomas.” However, the role of granulomas and the consequences of epithelioid transformation in mycobacterial infections remain unclear. Therefore, we hypothesize that genetic variants of the keratinization pathway, present in animals with multifocal lesions, induce the transformation of macrophages into EMs expressing CK, promoting resilience to PTB through the formation of ordered granulomas that prevent Map dissemination and limit tissue damage.

In this context, the present study aimed to investigate the keratinization pathway enrichment in cattle with multifocal lesions, and to analyze whether multifocally distributed EMs lead to the control of Map infection. For this purpose, the number and distribution pattern of EMs were investigated in distal jejunum (DJE) and distal jejunal lymph nodes (DJELN), focusing on granulomas from naturally infected animals with different pathological forms of PTB (focal, multifocal, diffuse intermediate, and diffuse multibacillary), and from negative controls with no visible lesions. Quantitative double-immunohistochemical (D-IHC) analysis was used, with ionized calcium-binding adapter molecule-1 (Iba1) and CK as markers of macrophages and epithelial cells, respectively. The potential role of CK and EMs in resilience to PTB was also analyzed.

## 2 Materials and methods

### 2.1 Ethics approval statement

Animals used in this study had their origin in commercial farms. All farmers were informed about the study and gave their consent and approval for the use of samples in the present study. This study was carried out in accordance with Directive 2012/63/EU of the European Parliament. Experimental procedures were evaluated by the SERIDA Animal Ethics Committee board approval and authorized by the Regional Consejería de Agroganadería y Recursos Autóctonos del Principado de Asturias, Spain (authorization codes PROAE 29/2015, PROAE 66/2019, and PROAE17/2022).

### 2.2 Animals and samples

Cattle were obtained from two collaborating farms located in the Principality of Asturias (Northwest of Spain), both with a known history of PTB. Animals were routinely tested for anti-Map antibodies using the IDEXX enzyme-linked immunosorbent assay (ELISA) and for *Mycobacterium bovis* by the tuberculin test as part of official control programs. Samples of blood, feces, and tissues were collected from all animals slaughtered in those farms due to PTB, reduced production yield, other diseases, or accidents. Blood samples were taken in serum clot activator Vacutainer® tubes (Vacuette, Kremsmunster, Austria) from the coccygeal vein and then transported to our laboratory at room temperature (RT). Serum was separated by centrifugation (2,500*g* for 20 min at RT) and then stored at −20 °C for subsequent ELISA analysis. Samples of feces were collected and either used directly for bacteriological culture or stored at −20 °C for subsequent real-time polymerase chain reaction (PCR) analysis. Tissue samples [DJE, ileocecal valve (ICV), and Ileocecal lymph nodes (ICVLN) and DJELN] were collected from slaughtered animals *in situ* at the local abattoir after evisceration. These samples were used for bacteriological culture, real-time PCR, histological classification, and D-IHC (DJE and DJELN). Positive bacteriological cultures were confirmed by Ziehl-Neelsen (ZN) analysis and conventional PCR.

The golden standard used for the classification of the animals included in this study was the type of histological lesions. Once classified, animals within each group were selected based on their infectious status so the groups were as homogeneous as possible.

A total of 28 Holstein Friesian cows (4 non-infected controls and 24 naturally infected animals, age range 0.81–10.39) were used for this study. The Map infection status of the 28 animals used in this study was determined by histopathology, specific detection of anti-Map antibodies in serum by ELISA (IDEXX, Montpellier, France), bacteriological culture, and real-time PCR of tissues and feces, following the procedures previously described (39).

### 2.3 Tissue preparation and histopathological classification of animals

Tissue samples were taken and processed using standard procedures. Samples were fixed in 10% neutral buffered formalin, sliced, and embedded in paraffin blocks. Tissue sections (4 μm) were cut, placed on microscope slides (Superfrost Plus, Menzel GmbH, Braunschweig, Germany), and dried at 37 °C for 24 h. Afterwards, tissue sections were stained by hematoxylin–eosin (H&E) and Ziehl-Neelsen (ZN) to evaluate lesions and confirm the presence of acid-fast bacteria. Representative images of ZN-positive sections can be visualized in Supplementary Figure S1. Slices were analyzed using an Olympus BH-2 light microscope (Olympus, Tokyo, Japan). Histological lesions associated with bovine PTB were classified according to Gonzalez et al. (11), examining four target sections (DJE, ICV, ICVLN, and DJELN). Focal lesions consist of small scattered and well-demarcated granulomas composed of macrophages and a few Langhans giant cells, located in DJELN and ICVLN, and within the lymphoid tissue associated with the lamina propria; the rest of the intestinal lamina propria is not affected. Multifocal lesions consist of numerous well-demarcated granulomas in the intestinal lymphoid tissues and the intestinal lamina propria. Diffuse lesions are characterized by extensive, severe inflammatory infiltrate with granulomas in the intestinal lymphoid tissues and lamina propria, which markedly alter the normal histological structure of the intestine, including submucosa, resulting in a clear merger of the villi. According to the inflammatory cell type present in the infiltrate and the number of acid-fast bacilli, diffuse lesions are further subdivided into diffuse lymphoplasmacytic paucibacillary, diffuse intermediate, and diffuse histiocytic multibacillary lesions (12).

As we were interested in investigating the enrichment of the keratinization pathway and the distribution pattern of EMs in granulomas, after histological classification (focal, multifocal, or diffuse) of the animals under study, the number of CK-expressing cells was quantified specifically in the granulomas by D-IHC. Initially, the four target tissues, DJE, ICV, ICVLN, and DJELN, were investigated to determine which one had the highest number of granulomas in the selected animals. We observed that in multifocal animals, the majority of the granulomas were present in DJE and DJELN. Focal animals did not have numerous granulomas in the DJE lymphoid tissue; they were more numerous in the lymph nodes, with a greater number in DJELN. The granulomas of animals with diffuse lesions were observed in abundant numbers in the four sections. Consequently, DJE and DJELN were selected for D-IHC, as these two sections showed higher numbers of granulomas. These two sections were considered to be representative of the host’s intestinal tissue response to Map infection. The DJE represents epithelial tissue, loose connective tissue (lamina propria), and associated lymphoid tissue. The addition of the DJELN samples to the experimental design was essential for quantification in older animals, as the extension of lymphoid-associated tissue (Peyer’s patches) in DJE sections is inversely correlated with the age of the animals within each group.

### 2.4 Single (S-IHC) and double-immunohistochemistry (D-IHC)

Prior to the D-IHC optimization, a single immunohistochemistry (S-IHC) assay was performed to evaluate the reactivity of the rabbit polyclonal anti-CK antibody in the experimental samples (DJE and DJELN), using bovine endometrium as a CK-positive epithelial control tissue (40, 41). A detailed description of both S-IHC and D-IHC procedures is provided in Supplementary Material.

For quantification of the number of CK-positive macrophages (EMs) within granulomas from animals with focal, multifocal, diffuse intermediate, and diffuse multibacillary lesions and in negative control animals with no visible lesions, the number of cells expressing both CK and Iba1 within the DJE and DJELN of each animal was investigated by sequential D-IHC (Supplementary Figure S2) *to detect simultaneously two different antigens, Iba1 and CK, in tissue samples*.

Formalin-fixed paraffin-embedded DJE and DJELN samples were cut into 3-μm sections and placed on microscope slides (Superfrost Plus, Menzel GmbH, Braunschweig, Germany). Sections were dewaxed and rehydrated using tap water at RT, and subjected to antigen retrieval using 0.1% trypsin (Sigma–Aldrich, St. Louis, MO, USA) dissolved in preheated tris-buffered saline (TBS) [5 mM tris (Merck KGaA, Darmstadt, Germany)/HCl (Panreac Química, SLU, Barcelona, Spain) pH 7.6, 136 mM NaCl (Merck KGaA, Darmstadt, Germany)] containing 0.1% CaCl_2_ (Merck, Darmstadt, Germany) pH: 7.8 for 45 min at 37 °C; Endogenous peroxidase activity was blocked with 3% hydrogen peroxide (Sigma–Aldrich, St. Louis, MO, USA) in methanol (VWR, Monroeville, PA, USA), 10 min at RT. Slides were washed with tap water at RT, and then, non-specific binding was blocked using 10% normal goat serum (Vector Laboratories) containing 3% bovine serum albumin (BSA, Sigma–Aldrich, St. Louis, MO, USA) for 15 min at RT.

For the first stain, tissue sections were incubated with rabbit polyclonal antibody anti-Iba1 (FUJIFILM Wako, Osaka, Japan) for specific macrophage identification (Iba1), which is largely restricted to cell of monocyte/macrophage lineage (42), at a 1:1,200 dilution overnight at 4 °C and then washed three times with TBS 1X at RT. After that, sections were incubated for 30 min at RT with a biotinylated anti-rabbit IgG secondary antibody produced in goat (Vector Laboratories, Burlingame, CA, USA) at a 1:200 dilution, and the slides were washed as previously described. For signal detection, sections were incubated for 30 min at RT with avidin–biotin complex [ABC kit Peroxidase (PO) Standard, Vector Laboratories] followed by three washes with 1X TBS and incubation with the PO substrate Diaminobenzidine tetrahydrochloride (DAB) for 2 min at RT. Afterwards, samples were rinsed with tap water for 5 min and washed with 1X TBS three times at RT. DAB was used as the first chromogen, as recommended (43), since its reaction product effectively masks and prevents non-specific cross-reactions in subsequent staining sequences.

For the second D-IHC stain, the same slides were incubated overnight at 4 °C with rabbit anti-cytokeratin polyclonal (Dako, CA, USA) at a 1:1,000 dilution. It is a wide-spectrum screening antibody that detects low-molecular-weight CKs (40–54 kDa), specifically KRT7-8 and KRT17-20 according to Moll’s designation, and high-molecular-weight CKs (48–67 kDa), specifically KRT1-6 and KRT9-16. After that, samples were washed three times with 1X TBS at RT. Bounded antibody was detected by incubation for 30 min at RT with alkaline phosphatase (AP)-conjugated goat anti-rabbit IgG secondary antibody (Sigma–Aldrich, St. Louis, MO, USA) followed by three washes with 1X TBS and the addition of the AP substrate 1-StepTM NBT/BCIP (Thermo Scientific, Rockford, USA) for 6 min at RT under microscopic control. Because the AP substrate (1-StepTM NBT/BCIP) and avidin–biotin PO substrate (DAB) can produce a widespread precipitate over the entire section, both reagents were freshly prepared and micropore filtered 0.22 μm (Merck Millipore, Cork, Ireland) immediately before application to the tissue.

Although the intestine and lymphoid tissue are primary locations of endogenous AP activity, no blocking was required in our experimental conditions. No endogenous AP activity was detected after 5, 10, and 20 min of NBT/BCIP incubation, in gut tissues and regional lymph nodes, using bovine endometrium as a positive control tissue (Supplementary Figure S3). This fact might be due to the antigen retrieval protocol used. It has been reported that AP enzymes from different species differ in their susceptibility to heat inactivation and exposure to 20% aqueous acetic acid dilutions (44).

Samples were rinsed with tap water for 5 min at RT and counterstained in Mayer’s hematoxylin (Merck KGaA, Darmstadt, Germany) for 5 s before washing, dehydrating, and mounting with DPX (Merck KGaA, Darmstadt, Germany). Single-Iba1-positive cells were stained brown (DAB), single-CK-positive cells were stained light blue (1-StepTM NBT/BCIP), and double Iba1/CK-positive cells were stained black. It is important to note that the results of the IHC should be interpreted with caution as the brown color in the S-IHC represents CK-positive cells and in the D-IHC Iba-1-positive cells.

The specificity of the immunoreagents used in the S-IHC and D-IHC was investigated by performing several controls (Table 1, Controls 1–3; Supplementary Material), showing that the anti-CK wide-spectrum screening antibody used exhibited specificity in the positive bovine endometrium control, and the biotinylated anti-rabbit IgG secondary antibody displayed no perceptible cross-reactivity with non-target proteins (Supplementary Figure S4). Specific CK staining was also observed by S-IHC in DJELN (Supplementary Figure S5) and DJE (Supplementary Figure S6).

**Table 1:** Positive and negative controls used in single and double immunohistochemistry to rigorously investigate immunoreagents’ specificity.

	Single immunohistochemistry	
Controls idendity	Primary antibody: anti-CK	Secondary antibody and avidin–biotin complex
Control 1 (BE)	Yes	Yes
Control 2 (BE)	No	Yes
Control 3 (DJE and DJELN)	No	Yes

	Double immunohistochemistry			
	First stain		Second stain	
	Primary antibody: anti-Iba1	PO-conjugated secondary antibody and avidin–biotin complex	Primary antibody: anti-CK	AP-conjugated secondary antibody
Control 4 (DJELN and DJE)	No	Yes	No	Yes
Control 5 (DJELN and DJE)	Yes	Yes	No	Yes
Control 6 (DJELN and DJE)	Yes	Yes	Yes	Yes

BE, bovine endometrium; DJE, distal jejunum; DJELN, distal jejunum lymph nodes; CK, cytokeratin; Iba1, ionized calcium-binding adapter molecule-1; Controls 1 and 2 for the single immunohistochemistry (S-IHC) were performed in BE, while Controls 3 (S-IHC) and 4–6 (double immunohistochemistry [D-IHC]) were carried out in both DJELN and DJE. For D-IHC, in the first stain, we used rabbit polyclonal anti-Iba1 as primary antibody and biotinylated goat anti-rabbit IgG as secondary antibody with avidin–biotin complex, and in the second stain, rabbit polyclonal anti-CK antibody and alkaline phosphatase (AP)-conjugated anti-rabbit IgG were used as primary and secondary antibodies, respectively.

Cross-reactivity and specificity of detection antibodies were also assessed by D-IHC of DJE and DJELN tissue samples, evaluating three controls (Controls 4–6 of Table 1). In the D-IHC, we expected to observe three types of positive immunolabeled cells (single-CK stained light blue, single-Iba1 stained brown, and double-Iba1/CK stained dark blue) and no immunolabeled cells. In the negative control (Control 4) performed with omission of the two primary antibodies (anti-Iba1 and anti-CK) no positive cells were observed (Supplementary Figure S7A,D for DJELN and Supplementary Figures S8A,D for DJE) after 3 min incubation with the AP substrate, indicating that: (1) secondary antibodies did not display cross-reactivity with non-target proteins; (2) any endogenous PO and AP activity had been correctly inactivated; and (3) PO and AP substrates did not show unspecific reactivity. In the negative control carried out with omission of the anti-CK primary antibody used in the second stain (Control 5) no single-CK (light blue) or double-Iba1/CK positive cells (dark blue) were observed (Supplementary Figures S7B,E for DJELN and Supplementary Figures S8B,E for DJE), indicating that in the sequential D-IHC procedure design the goat anti-rabbit IgG AP-conjugated secondary antibody did not bind unspecifically to anti-Iba1 primary antibodies; no cross-reactivity was detected between the two staining sequences. In the D-IHC positive control (Control 6), carried out without omission of any reactive, the three types of positive cells were observed within the granuloma (Supplementary Figures S7C,F for DJELN and Supplementary Figures S8C,F for DJE). In the DJE positive control, some single-CK light blue immunostaining was observed outside of the granulomas in the villi as part of the normal characteristic expression of CK in the intestine (Supplementary Figure S8). These results demonstrate that the procedure of the D-IHC worked well in both tissue types, with no cross-reactivity observed between detection antibodies.

Variations in the brown signal intensity were occasionally observed in “macrophages” between the different images shown in the figures. This might be explained by the type of IHC (single or double) they have been submitted to, the magnification used (the color in 1,000× images is less intense than at 400×) or the activation stage of the macrophages. There are multiple technical factors inherent to the D-IHC protocol that might be responsible for the variations observed in the intensity of the brown immunohistochemical signal (DAB-stained cells) across different images. Specifically, the antigen retrieval method used, this unmasking approach, though effective for CK, may produce variable epitope exposure for Iba-1, potentially leading to reduced signal intensity in some cells. Additionally, during the optimization of the D-IHC protocol, a milder staining intensity was targeted for the Iba1 signal to ensure clear visualization of both markers within the same tissue section and to facilitate manual quantification.

### 2.5 Image acquisition and quantification

Immunolabeled sections from D-IHC were observed using an Olympus BH-2 light microscope (Olympus, Tokyo, Japan). Individual images were acquired using an Olympus DP-12 digital camera (Olympus, Tokyo, Japan). Images were taken throughout histological sections, selecting non-overlapping fields with granulomatous lesions, avoiding areas containing preparation artefacts, cell debris, or the edges of the slide. In the case of control animals, although granulomas are not present, counting was carried out in equivalent regions of the preparations, in other words, the images were taken in areas where according to the lesion classification of Gonzalez et al. (11) granuloma formation usually occurs in the different lesion types described, such as the cortical and paracortical area in DJELN, and the apical area of the villi and lymphoid/MALT tissue in DJE. To minimize selection bias, fields were chosen blindly with respect to the infection status and lesion type of the sample. The microscope stage was moved in a predetermined pattern in all granuloma areas of the samples (systematic random sampling).

To evaluate cytokeratin expression in cattle with different histopathological forms of PTB, the number of single and double positive stained cells within granulomatous lesions and controls in each tissue sample from each individual was counted in 10 randomly selected fields per individual at a final magnification of 400×, so a total of 40–80 fields were examined for each histopathological type. Each field corresponded to one image captured using a 40× objective (1,600 × 1,200 pixels) having a theoretical optical system area of 41207.52 μm^2^ (234.4 μm length × 175.8 μm height). The number of positive immunolabeled cells was manually counted in each selected field/image using the ImageJ program (National Institutes of Health, USA). Cell counts were performed on anonymized images to reduce observer bias. Results are expressed as a mean number of positive cells per field ± standard deviation (SD) for each histopathological group. Positive cells in the D-IHC were classified as single-CK positive cells, single-Iba1 positive cells, and double-Iba1/CK positive cells.

### 2.6 Statistical analysis

Statistical analysis was carried out using the R program (R Development Core Team, version 4.1.3). Differences of quantitative variables between two groups were carried out using the Student’s *t*-test or the Wilcoxon test for independent samples, depending on the normality hypothesis. Differences between three or more groups were analyzed using the analysis of variance (ANOVA) or Kruskal–Wallis test, depending on whether the hypotheses of homoscedasticity and normality were verified. When the Kruskal–Wallis test and the ANOVA test were statistically significant, the non-parametric *post hoc* Dunn’s test and the parametric *post hoc* Tukey’s test, respectively, were conducted to determine exactly which pairwise groups had statistically significant differences. Tukey’s Honestly Significant Difference (HSD) (parametric) and Holm-Bonferroni method (non-parametric) were used for correcting Type I error when performing multiple comparisons. The level of significance used was 0.05.

## 3 Results

### 3.1 Map infection status of studied animals

The Map infection status of the 28 animals used in this study is shown in Table 2. The animals were classified into five groups according to the presence/absence and type of histological lesions in gut tissues (11, 12) (Table 2): (1) the healthy control group (*n* = 4, age range 0.81 to 3.26, mean 2.01 ± 1.16) consisted of animals with no visible lesions, negative by ELISA, and bacteriological culture and real-time PCR of tissues and feces. We found very few control animals without lesions observed (only 5% of the total number of cows diagnosed in our laboratory are controls); the high prevalence of paratuberculosis in cattle in our region makes it challenging to identify adequate control animals, especially among older cattle, as they are more likely to have been exposed to Map infection; (2) the focal group (*n* = 7, age range 2.12–0.48, mean 6.53 ± 2.35) consisted of animals with focal lesions, all ZN positive and ELISA negative; (3) the multifocal group (*n* = 5, age range 2.48–9.67, mean 6.42 ± 2.66) included animals with multifocal lesions, positive by at least one of the diagnostic tests used, with low bacterial load (ZN technique) and without clinical signs; (4) the diffuse intermediate group (*n* = 6, age range 4.47–7.02, mean 5.46 ± 0.91) included animals with diffuse intermediate lesions with all animals positive by ELISA and PCR of feces and tissues, more abundant mycobacteria observed in the ZN and 83% of the animals showing clinical signs; and (5) the multibacillary diffuse group (*n* = 6, age range 2.92 to 10.39, mean 5.90 ± 2.54), which was composed of animals with multibacillary diffuse lesions with a large number of Map bacteria present, with 100% of the animals positive by ELISA and with clinical signs. Clinical signs were observed in 0, 0, and 91.66% of the animals with focal, multifocal, and diffuse (intermediate and multibacillary) lesions (no information was available for three focal and two multifocal animals), respectively. No gross or histologic lesions compatible with other inflammatory processes were identified during post-mortem inspection of the studied animals.

**Table 2:** Infection status of the animals used in this study classified according to the type of paratuberculosis-associated histological lesions present in their gut tissues and regional lymph nodes.

Histological group	Animal ID	Age (years)	Clinical signs	ZN	Anti-Map ELISA	PCR faeces	PCR tissues	Culture faeces	Culture tissues
Focal	29 N	8.44	ND	+	NEG (3.07)	NC	POS (39.85)	NEG	NEG
	36 N	7.36	ND	+	NEG (7.92)	NEG	POS (39.23)	NEG	POS
	51	9.48	ND	+	NEG (2.79)	NEG	POS (29.38)	NEG	NEG
	89	6.45	NO	+	NEG (1.48)	NEG	POS (32.98)	NEG	NEG
	109	5.85	NO	+	NEG (5.08)	NEG	POS (34.14)	NEG	NEG
	157	6.02	NO	+	NEG (1.90)	POS (39.15)	POS (35.23)	NEG	NEG
	160	2.12	NO	+	NEG (2.37)	NEG	NEG	NEG	POS
Multifocal	6 N	6.58	ND	+	NEG (6.57)	NC	NEG	NEG	POS
	27	5.65	ND	+	POS (148.65)	POS (33.60)	POS (24.38)	NEG	NEG
	48	9.67	NO	+	NEG (4.82)	NEG	NEG	NEG	POS
	65	7.7	NO	+	NEG (3.46)	NC	POS (28.97)	NEG	NEG
	213	2.48	NO	+	NEG (3.12)	NEG	POS (37.35)	NEG	NEG
Diffuse intermediate	5	5.22	YES	++	POS (205.94)	POS (28.84)	POS (21.63)	NEG	POS
	32	4.47	YES	+	POS (241.18)	POS (31.27)	POS (25.65)	POS (>50)	POS
	59	7.02	YES	++	POS (288.75)	POS (33.43)	POS (21.23)	NEG	POS
	68	6.01	YES	++	POS (245.72)	POS (22.54)	POS (17.05)	POS (>50)	POS
	87	5.09	YES	++	POS (113.49)	POS (28.20)	POS (29.42)	NEG	NEG
	125	4.94	NO	+	POS (211.41)	POS (35.13)	POS (28.13)	NEG	POS
Diffuse multibacillary	88	2.92	YES	+++	POS (284.51)	POS (23.83)	POS (22.90)	NEG	POS
	92	6.70	YES	+++	POS (174.43)	POS (38.33)	POS(34.36)	POS (>50)	POS
	99	4.82	YES	+++	POS (157.83)	POS (28.14)	POS (29.05)	POS (>50)	POS
	101	5.82	YES	+++	POS (242.36)	POS (29.39)	POS (19.19)	POS (>50)	POS
	103	10.39	YES	+++	POS (155.82)	POS (30.23)	POS (23.65)	NEG	POS
	115	4.74	YES	+++	POS (215.95)	POS (23.07)	POS (17.64)	NEG	POS
Control group without lesions	4 N	3.26	ND	−	NEG (5.44)	NEG	NEG	NEG	NEG
	13 N	0.81	ND	−	NEG (8.84)	NEG	NEG	NEG	NEG
	94	2.70	NO	−	NEG (1.26)	NEG	NEG	NEG	NEG
	113	1.27	NO	−	NEG (2.45)	NEG	NEG	NEG	NEG

ID, cows’ identification; ND, unknown; the number of + shown in parenthesis in the Ziehl–Neelsen (ZN) column indicates the amount of acid–alcohol resistant bacteria present; ZN results correspond to the most positive sample of the four tissue sections examined (distal jejunum, ileocecal valve, ileocecal lymph node, and distal jejunal lymph node); Map, refers to *Mycobacterium avium* subsp. *paratuberculosis*; In the anti-Map ELISA column, the numerical value obtained in the ELISA is shown in parentheses; the number shown in parentheses in the PCR columns corresponds to the cycle threshold (Ct) obtained in the amplification of the F57 sequence; NC, non-conclusive result; >50 refers to the number of colonies isolated in the bacteriological culture.

### 3.2 Morphological analysis, distribution, and patterns of Iba1 and CK expressing cells in DJELN and DJE of cattle with different histopathological forms of bovine paratuberculosis

The enrichment of the keratinization pathway in cattle with multifocal lesions was investigated within granulomas of DJELN and DJE samples, by double-Iba1/CK immunohistochemistry and quantification of the number of CK-positive cells in animals with different pathological forms of PTB (*n* = 24) and control animals without lesions (*n* = 4).

**Figure 1:**
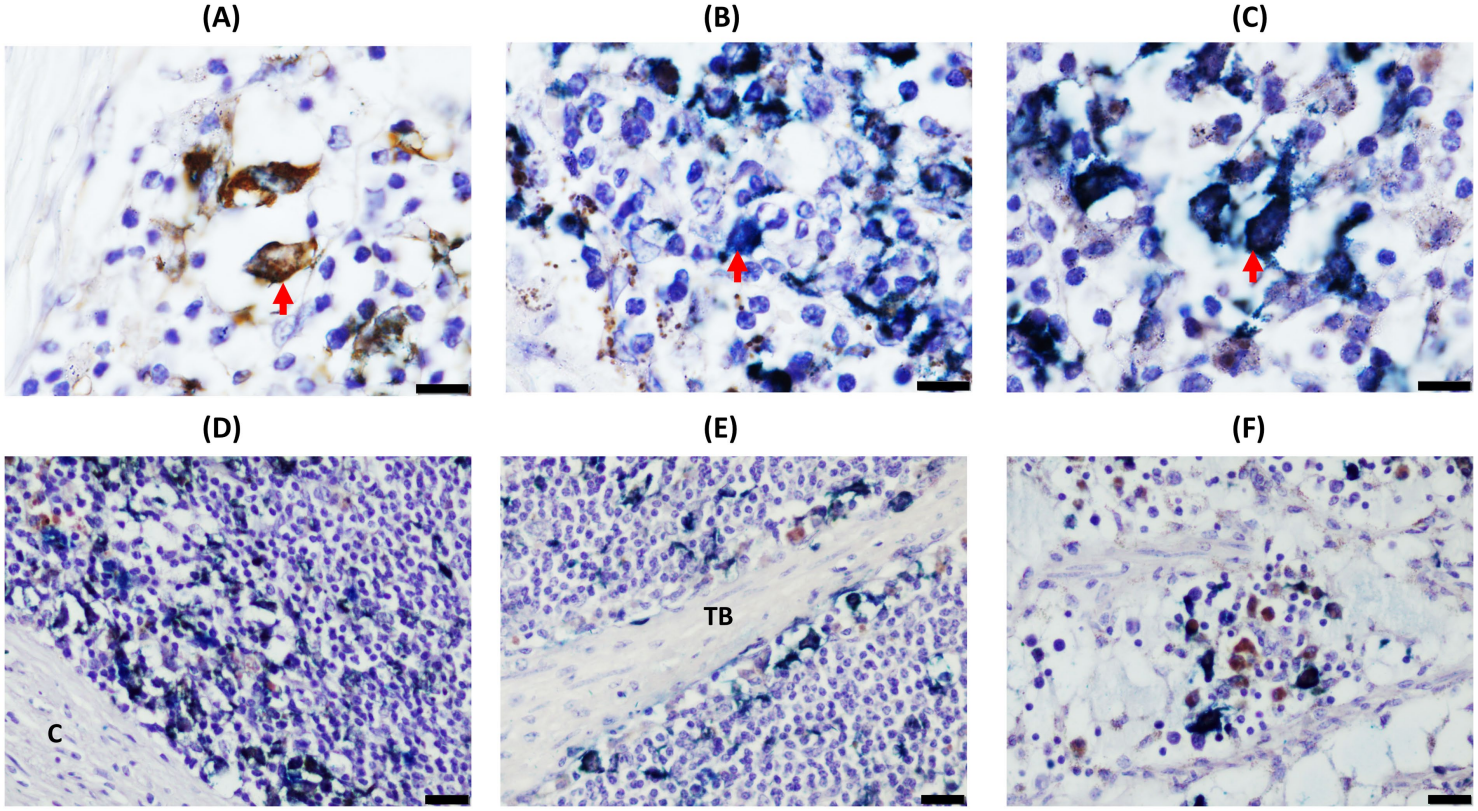
Double-ionized calcium-binding adapter molecule-1 (Iba1)/cytokeratin (CK) immunohistochemical analysis of distal jejunal lymph node (DJELN) samples of a control cow without lesions showing the distribution and morphological appearance of the three immunolabeled cell types detected (CK-positive, Iba1-positive, and double-Iba1/CK-positive cells). Images in **(A–C)** show the different immunolabeled cell types: **(A)** single Iba1-positive cells (brown color) in the cortex; **(B)** single CK-positive cells (bright blue) in medulla; and **(C)** double Iba1/CK-positive cells (dark blue) in the cortex. Images in **(D–F)** display different areas of distribution of the three cell types: **(D)** cortex area of DJELN close to the line of the subcapsular sinus; **(E)** trabeculae at the height of the interfollicular cortex of DJELN; **(F)** medullary area of DJELN. **(A–C)** magnification 1,000×, bars 10 μm. **(D–F)** magnification 400×, bars 20 μm. Red arrows point to single cells representing the three different immunolabeled cell types. C, capsule; TB, trabeculae.

**Figure 2:**
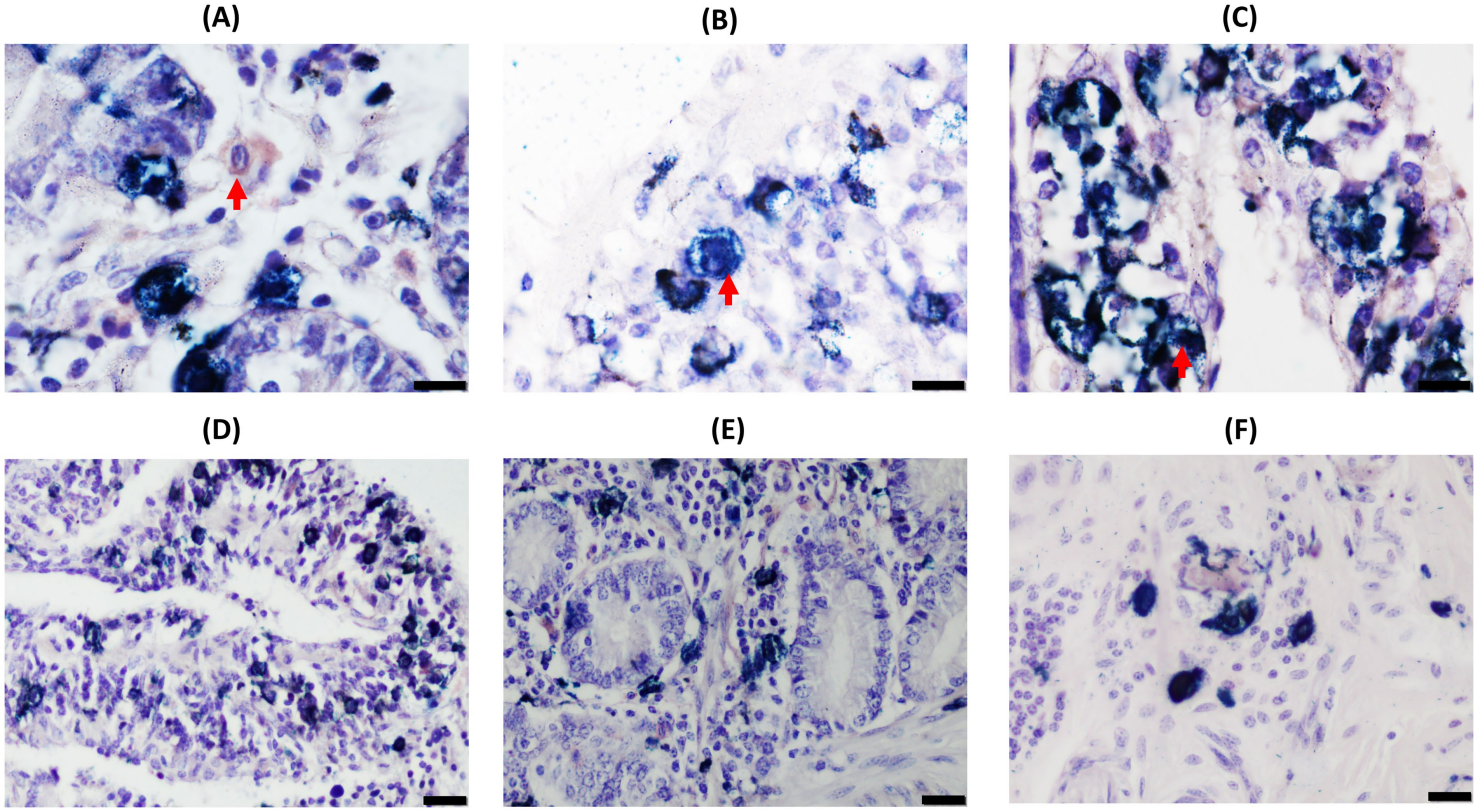
Double-ionized calcium-binding adapter molecule-1 (Iba1)/cytokeratin (CK) immunohistochemical analysis of distal jejunum (DJE) samples of a control cow without lesions showing the distribution and morphological appearance of the three immunolabeled cell types detected (CK-positive, Iba1-positive, and double-Iba1/CK-positive cells). Images in **(A–C)** show the different immunolabeled cell types: **(A)** single Iba1-positive cells (brown cells) in the basal mucosa; **(B)** single CK-positive cells (bright blue) in the apical mucosa; **(C)** double Iba1/CK-positive cells (dark blue) in the apical mucosa. Images in **(D–F)** display different areas of distribution of the three cell types in DJE: **(D)** apical area of DJE mucosa; **(E)** basal area of DJE mucosa; **(F)** DJE submucosa. **(A–C)** magnification 1,000×, bars 10 μm. **(D–F)** magnification 400×, bars 20 μm. Red arrows point to single cells representing the three different immunolabeled cell types.

D-IHC analysis showed four types of cells within the granuloma and around the structure of both DJELN and DJE samples (Figures 1, 2, respectively): single-Iba1 positive macrophages stained as brown cells (Figure 1A for DJELN and Figure 2A for DJE), single-CK positive cells detected as bright blue cells (Figure 1B for DJELN and 2B for DJE), double-Iba1/CK positive cells observed as dark blue or black cells (Figure 1C for DJELN and 2C for DJE), and negative cells (no immunolabeled cells).

**Figure 3:**
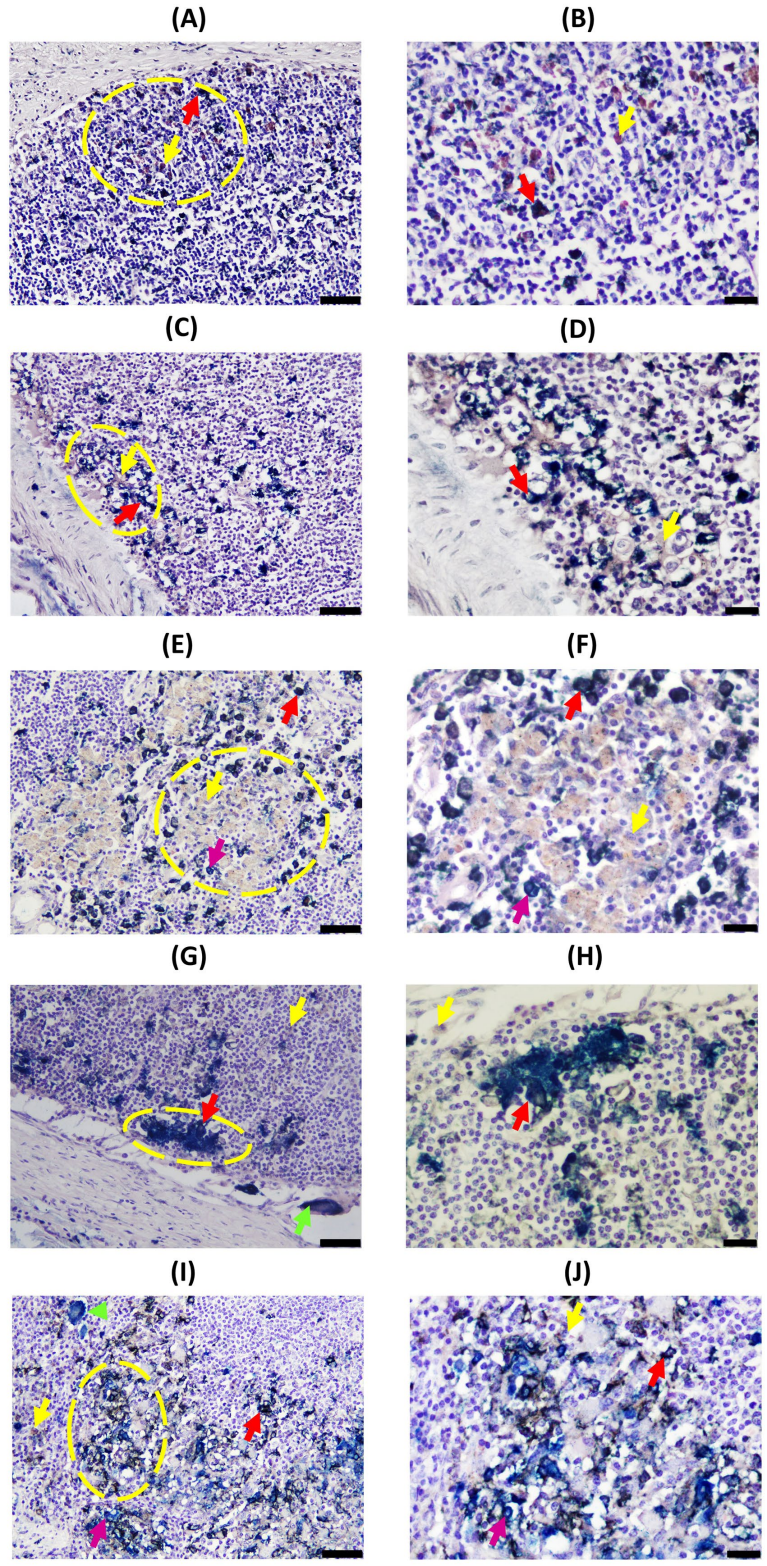
Representative images of cytokeratin (CK) and Iba1 double-immunohistochemical (D-IHC) analysis of jejunal lymph nodes (DJELN) samples of animals with different histopathological forms of PTB and control animals without lesions. **(A,B)** Control cow with no lesions detected; **(C,D)**, **(E,F)**, **(G,H)**, and **(I,J)** show granulomas in animals with focal, multifocal, diffuse intermediate, and diffuse multibacillary lesions in their intestinal tissues, respectively. The bars represent 50 μm, and sections were examined at 200× magnification. Yellow circles indicate areas shown at a higher magnification in images **(B)**, **(D)**, **(F)**, **(H)**, and **(J)**. The bars represent 20 μm. CK and Iba1 immunolocalization was investigated using a broad-spectrum rabbit polyclonal anti-CK antibody (Dako, CA, USA) and a rabbit polyclonal anti-Iba1 antibody (FUJIFILM Wako, Osaka, Japan). All granulomas depicted belong to the most DJELN cortical zone. Red arrows point to examples of double-stained cells (dark blue or black); pink arrows point to single CK-positive cells (light blue); yellow arrows point to single Iba1-positive cells (brown color); and green arrows point to multinucleated giant cells. Notice that CK-expression in animals with multifocal lesions showed a pattern where epithelioid macrophages (EMs) are located around the granuloma **(E,F)**, while in animals with diffuse lesions, a different pattern is observed where EMs are distributed through the whole granuloma **(G,H)**.

In DJELNs (Figures 1, 3), both expected CK-positive cell types (single-CK and double-Iba1/CK positive cells) were observed in all the samples (*n* = 28), including the control samples. No morphological differences were observed in these cell types between animals of different histopathological groups and ages. Single-CK immunolabeled cells (bright blue) in the DJELN were observed in a highly variable number as part of the granuloma and scattered in the cortex and sometimes near blood vessels, supporting tissue and trabeculae (TB) and medullary area. Single-CK positive cells had medium to large-sized nuclei (4.22–8.65 μm in diameter) with a semi-round shape and sparse cytoplasm compatible in morphology and localization with reticular cells (RCs) (Figure 1B). Single-Iba1 positive cells (brown cells) presented large round to oval nuclei (5.11–9.83 μm in diameter) and abundant cytoplasm (Figure 1A) and had a similar appearance and distribution to tissue macrophages. Double-Iba1/CK positive cells, which are macrophages expressing CK, had large round to oval nuclei (4.36–9.12 μm in diameter) and abundant cytoplasm, and were identified as EMs. These double-immunolabeled cells were in the cortex of the lymph nodes in a scattered manner (Figure 1D), around the lymphoid follicles, as well as close to the germinal center and in the subcapsular sinus. These cells were also observed in the paracortex area and especially in the medullary area, tending to be more numerous toward the efferent lymphatic vessels in both infected and uninfected animals (Supplementary Figures S7C,F). Control animals without lesions showed a similar pattern of CK-expression to that mentioned above (Figures 3A,B); however, as the severity of the lesions increases, the number of double-Iba1/CK positive cells seems to increase in the cortex area, showing two different patterns. In pattern 1, double-Iba1/CK EMs were found surrounding the granuloma with abundant single Iba1 foamy macrophages and a few single-CK cells inside the granuloma, while in pattern 2, double Iba1/CK EMs were within the granuloma, forming the granuloma itself, accompanied by a few single CK cells with no single-Iba1 foamy macrophages observed (Supplementary Figure S9). The first scenario was more common in animals with focal and multifocal lesions (Figures 3C–F), while the second one seemed to be more frequent in animals with diffuse intermediate and diffuse multibacillary lesions (Figures 3G–J). Multinucleated giant cells, both CK-positive (Figures 3G,I) and CK-negative, were also observed in DJELN, both outside and forming part of granulomas.

**Figure 4:**
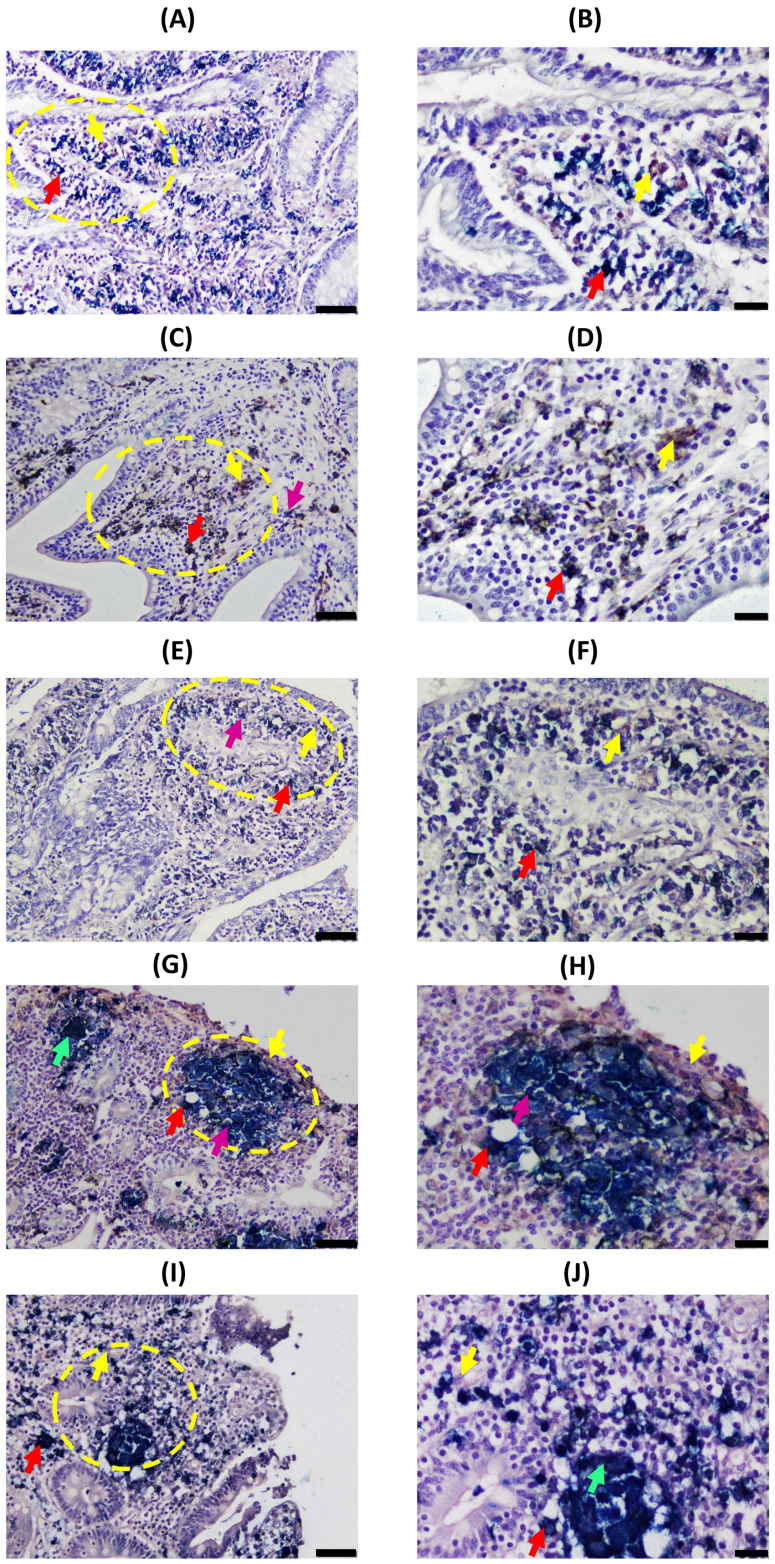
Representative images of Cytokeratin (CK) and Iba1 double-immunohistochemical (D-IHC) results in distal jejunum (DJE) of infected cows with different histopathological forms of PTB and control cows without observed lesions. **(A,B)** Control cow with no detected lesions; **(C,D)**, **(E,F)**, **(G,H)**, and **(I,J)** show granulomas in animals with focal, multifocal, diffuse intermediate, and diffuse multibacillary lesions in their intestinal tissues, respectively. The bars represent 50 μm, and sections were examined at 200× magnification. Yellow circles indicate the areas that are shown in the right-hand column at a higher magnification (400×) corresponding to images **(B)**, **(D)**, **(F)**, **(H)**, and **(J)**. The bars represent 20 μm. All granulomas shown are located in the most DJE apical zone. Red arrows point to examples of double-stained cells (dark blue or black); yellow arrows point to single Iba1-positive cells (brown color); pink arrows point to examples of single-CK-stained cells (bright blue); and green arrows point to multinucleated giant cells. CK and Iba1 immunolocalization was investigated using a broad-spectrum rabbit polyclonal anti-CK antibody (Dako, CA, USA) and a rabbit polyclonal anti-Iba1 antibody (FUJIFILM Wako, Osaka, Japan). Notice that CK-expression in animals with multifocal lesions shows a pattern where epithelioid macrophages (EMs) are located around the granuloma **(E,F)**, while in animals with diffuse lesions, a different pattern is observed where EMs are distributed through the whole granuloma **(G–J)**.

In DJE (Figures 2, 4), single-Iba1, single-CK, and double-Iba1/CK positive cells were also observed in all animals. Single Iba1-positive cells (brown cells) presented large, round to oval nuclei and abundant cytoplasm and had a similar appearance to tissue macrophages distributed throughout the lamina propria. Single CK-stained cells had medium- to large-sized round nuclei with scarce cytoplasm (Figure 2B). These cells were observed in a highly variable number as part of the granulomas and scattered in the mucosa (Figures 2D,E) and submucosa (Figure 2F) of DJE. Double-Iba1/CK positive, with large round to oval nuclei and abundant cytoplasm (Figure 2C), were located scattered throughout the lamina propria, mostly in the apical area (Figure 2D), around and in the crypts of Lieberkühn (Figure 2E), surrounding the villi (Figures 4B–D). As in the case of DJELN samples, control animals without lesions did not show a striking pattern of CK expression (Figures 4A,B). Still, it appeared that as the severity of the lesion increased. Hence, it fixed the number of double-Iba1/CK positive cells in the granuloma area with granulomas showing the same two patterns previously described for DJELN (Figure 4F where double-Iba1/CK positive cells are found surrounding the granuloma, and Figure 4H, where they were within the granuloma).

### 3.3 Evaluation of CK expression in granulomatous lesions of cattle with different histopathological forms of bovine paratuberculosis

The results of the quantification of the number of single and total-Iba1 (single and double Iba1 positive cells), single and total-CK (single and double CK), and double-Iba1/CK positive cells in DJELN are shown in Table 3. With respect to the number of single-CK positive cells (cells expressing cytokeratin that are not apparently macrophages, compatible with RCs) per field and histological group, significant differences were observed between the different histopathological groups (Kruskal–Wallis test, *p* < 0.001). Specifically, *post hoc* Dunn’s test reported differences between the medians of the multifocal group [7.00 (2.00–14.00)] and the control [2.00 (0.00–4.25)] groups (*p* = 0.002). Regarding single-Iba1 positive cells (macrophages expressing Iba1 but not CK), no significant differences were observed between groups (Kruskal–Wallis, *p*-value = 0.07). As for the mean number of double-immunolabeled cells (EMs) per field, the group of animals with multifocal lesions showed the highest mean number (49.68 ± 24.99) and the focal group the lowest (31.14 ± 19.52). In this case, significant differences were observed between the cows with multifocal lesions and the cows with focal, diffuse intermediate, diffuse lesions (including intermediate and multibacillary) and controls (Tukey’s test, *p* < 0.001, *p* = 0.015, *p* = 0.038, and *p* < 0.001, respectively). The multifocal group also showed higher numbers of double-immunolabeled cells than the diffuse multibacillary group; however, no significant differences were observed in this case (*p* = 0.513). Regarding the total number of CK-expressing cells (single and double), significant differences between the multifocal and the focal (*p* < 0.001), and the control group (*p* < 0.001), were observed. The multifocal group had higher numbers of CK-positive cells than the diffuse group; however, no significant differences were observed (*p* = 0.106). No significant differences were observed in the total number (single and double) of Iba1-positive cells between groups (ANOVA, *p*-value = 0.389). Significant differences in the mean values of single-CK, double-Iba1/CK and total CK-expressing cells were also observed between infected animals (focal, multifocal and diffuse animals) and control animals without lesions (Welch’s test *p* < 0.001, Student’s test *p* = 0.042 and Student’s test *p* = 0.004, respectively), showing the control animals lower mean numbers than the infected animals. Control animals showed lower numbers of total Iba1 positive cells than infected animals, although differences were not significant (*p* = 0.091).

**Table 3:** Quantification of cytokeratin and Iba1 expressing cells in jejunal lymph nodes granulomatous lesions of cattle with different histopathological forms of bovine paratuberculosis.

		Distal jejunal lymph nodes				
Histopathological group	N	Single-CK+ cells	Single-Iba1 + cells	Double-Iba1/CK + cells	Total (single and double-CK + cells)	Total (single and double-Iba1 + cells)
Focal	70	3.50 (0.25–7.75)^b,c^	69.00 (52.25–100.75)^a^	31.14 ± 19.52^a^	34.00 (19.25–52.75)^a,e^	106.71 ± 38.30^a^
Multifocal	**50**	**7.00 (2.00–14.00)** ^**b,d**^	**56.50 (42.00–74.50)** ^**a**^	**49.68 ± 24.99** ^**b,d**^	**55.00 (33.75–81.75)** ^**b,d**^	**109.36 ± 39.63** ^**a**^
Diffuse	120	6.00 (2.00–13.00)^d^	72.00 (50.75–88.25)^a^	39.58 ± 22.86^a^	51.00 (25.50–67.50)^d^	111.31 ± 35.01^a^
Diffuse intermediate	60	6.00 (2.00–11.25)^b^	69.50 (47.00–95.75)^a^	36.20 ± 24.19^a,c^	42.50 (21.00–66.00)^a,b,d,f^	108.57 ± 36.73^a^
Diffuse multibacillary	60	6.50 (2.00–15.50)^b^	73.50 (55.50–83.25)^a^	42.97 ± 21.10^c,d^	53.00 (35.75–70.00)^b,d^	114.05 ± 33.28^a^
Controls without lesions	40	2.00 (0.00–4.25)^a,c^	70.00 (37.00–91.50)^a^	31.20 ± 21.59^a,c^	32.00 (18.00–48.25)^c,e,f^	98.62 ± 42.56^a^
Test used for statistical analysis multiple groups		Krustal-Wallis (*p* < 0.001)	Krustal-Wallis (*p* = 0.07)	ANOVA (*p* < 0.001)	Krustal-Wallis (*p* < 0.001)	ANOVA (*p* = 0.389)

Post hoc		Dunn’s test		Tukey’s test	Dunn’s test	
Group	N	Single-CK+ cells	Single-Iba1 + cells	Double-Iba1/CK + cells	Total (single and double-CK + cells)	Total (single and double-Iba1 + cells)
Controls without lesions	40	3.1 ± 4.51	67.42 ± 34.40	31.20 ± 21.59	34.00 ± 22.43	98.62 ± 42.56
Infected animals with lesions	240	8.70 ± 10.89***	70.34 ± 30.16	39.23 ± 23.24*	47.92 ± 28.60**	109.56 ± 36.88
Test used for statistical analysis of two groups		Welch’s test (*p* < 0.001)	Student’s test (*p* = 0.58)	Student’s test (*p* = 0.042)	Student’s test (*p* = 0.004)	Student’s test (*p* = 0.091)

CK, cytokeratin; Iba1, ionized calcium-binding adapter molecule 1; +, positive; N, number of fields examined per histopathological group. Results are expressed as mean or median values of the number of immunolabeled cells per field and histopathological group, depending on the statistical test used to analyse the data (mean ± standard deviation for parametric tests and median [interquartile range P25–P75] for non-parametric tests). Superscript letters indicate the results of the complete *post hoc* analysis (i.e., all possible pairwise comparisons). Groups with the same superscript letter are not significantly different from each other. Groups with different letters show statistically significant differences. Asterisks indicate whether the differences between infected and control animals are significant (*p-value < 0.05; **p-value < 0.01; ***p-value < 0.001). Results obtained for the multifocal group are shown in boldface.

The levels of cytokeratin expression in granulomas of cattle with different histopathological forms of bovine paratuberculosis were also evaluated in DJE samples (Table 4). Regarding single-CK positive cells, significant differences were found between infected animals (focal, multifocal, and diffuse) and control animals (Test of Welch, *p*-value = 0.047) and between the multifocal group and the focal (Dunn’s test, *p*-value = 0.003) and control (Dunn’s test, *p*-value = 0.036) groups, showing the multifocal group had the highest numbers of immunolabeled cells. However, no significant differences between pairs of histopathological groups were observed for single-Iba1 positive cells, double-immunolabeled cells, total number of CK, and total number of Iba1-immunolabeled cells.

**Table 4:** Quantification of cytokeratin and Iba1 expressing cells in distal jejunum granulomatous lesions of cattle with different histopathological forms of bovine paratuberculosis.

		Distal jejunum				
Histopathological group	N	Single-CK+ cells	Single-Iba1 + cells	Double-Iba1/CK + cells	Total (single and double-CK + cells)	Total (single and double-Iba1 + cells)
Focal	70	7.00 (1.00–13.00)^a,c^	59.11 ± 25.60^a^	53.00 (35.25–70.75)^a^	62.00 (43.25–79.75)^a^	113.96 ± 40.94^a^
Multifocal	**70**	**12.50 (3.00–34.50)** ^**b**^	**46.22 ± 25.45** ^**a**^	**47.00 (28.00–86.75)** ^**a**^	**73.50 (33.75–118.75)** ^**a**^	**107.40 ± 39.14** ^**a**^
Diffuse	130	10.00 (4.75–19.00)^b^	57.29 ± 30.99^a^	62.00 (41.50–84.25)^a^	75.00 (50.50–103.75)^a^	121.52 ± 45.53^a^
Diffuse intermediate	70	10.00 (4.00–18.25)^a,b,c^	58.88 ± 32.96^a^	63.00 (34.50–96.25)^a^	76.50 (41.00–113.50)^a^	125.78 ± 52.26^a^
Diffuse multibacillary	60	10.00 (5.00–19.00)^a,b,c^	55.70 ± 29.07^a^	57.00 (45.75–78.50)^a^	73.50 (53.75–99.50)^a^	117.25 ± 37.59^a^
Controls without lesions	50	7.00 (0.00–13.25)^c^	50.42 ± 28.02^a^	52.50 (42.50–72.00)^a^	63.50 (44.00–86.25)^a^	106.85 ± 45.35^a^
Test used for statistical analysis multiple groups		Krustal-Wallis (*p* = 0.001)	ANOVA (*p* = 0.08)	Krustal-Wallis (*p* = 0.438)	Krustal-Wallis (*p* = 0.193)	ANOVA (*p* = 0.145)

Post hoc test		Dunn’s test				
Group	N	Single-CK+ cells	Single-Iba1 + cells	Double-Iba1/CK + cells	Total (single and double-CK + cells)	Total (single and double-Iba1 + cells)
Controls without lesions	50	9.55 ± 11.27	50.42 ± 28.02	56.42 ± 24.71	65.97 ± 30.18	106.85 ± 45.35
Infected animals with lesions	270	13.68 ± 15.25*	55.52 ± 28.70	60.85 ± 35.88	74.53 ± 42.16	116.37 ± 43.15
Test used for statistical analysis of two groups		Welch’s test (*p* = 0.047)	Student’s test (*p* = 0.298)	Welch’s test (*p* = 0.333)	Welch’s test (*p* = 0.124)	Student’s test (*p* = 0.201)

CK, cytokeratin; Iba1, ionized calcium-binding adapter molecule-1; +, positive; N, number of fields examined per histopathological group. Results are expressed as mean or median values of the number of immunolabeled cells per field and histopathological group, depending on the statistical test used to analyze the data (mean ± standard deviation for parametric tests and median [interquartile range P25–P75] for non-parametric tests). The superscript letters indicate the result of the complete *post hoc* analysis (i.e., all possible pairwise comparisons). Groups with the same superscript letter are not significantly different from each other. Groups with different letters show statistically significant differences. Asterisks indicate whether the differences between infected and control animals are significant (*p-value < 0.05). Results obtained for the multifocal group are shown in boldface.

## 4 Discussion

The knowledge of the dynamic composition and structure of the granuloma, made up of macrophages in different stages of differentiation and other immune cells, may help to understand the role of the granuloma in the control of Map infection.

To the best of our knowledge, this is the first study to analyze the number and distribution pattern of CK-expressing cells in granulomas from cattle with different pathological forms of PTB, with the aim of investigating the potential role of EMs in resilience and Map infection control. A limitation of this study is the relatively small number of animals included, particularly in the multifocal lesion and healthy subgroup (*n* = 5 and 4, respectively), which may reduce statistical power and limit generalizability.

Our results showed that CK expression was enriched in granulomas of cattle with multifocal lesions. Animals with multifocal lesions had significantly higher numbers of double Iba1/CK positive EMs in DJELN granulomas and slightly higher numbers of single-CK expressing cells in DJELN and DJE granulomas. These observations align with the study of Canive et al. (29), who reported an enrichment of the keratinization pathway in cattle with multifocal lesions. The relatively high macrophage numbers in the controls were expected, given the abundance of tissue macrophages in the gastrointestinal system (45), while the high number of EMs in the control group may reflect prior exposure to Map or other immunological stimuli that influence epithelioid transformation (46).

Regarding EMs, two different distribution patterns were observed (Supplementary Figure S9). The majority of granulomas in multifocal animals presented an ordered structure, where EMs tended to form a barrier surrounding the granuloma, which was associated with lower Map loads and the absence of clinical signs (Table 2). These findings suggested that CK-expressing EMs may contribute to granuloma organization and stabilization in animals with multifocal lesions, potentially limiting bacterial spread and protecting surrounding tissue. This barrier-like organization may represent a potential resilience mechanism that would hypothetically control PTB progression. In contrast, animals with diffuse lesions did not show a barrier-like arrangement; they had EMs mainly throughout the granuloma, higher Map loads, and clinical signs. These animals failed to contain the infection efficiently despite having high titers of anti-Map antibodies (Table 2). Thus, both the number and distribution pattern of EMs appear to be crucial factors to control Map load and to avoid the development of clinical disease. Although animals with focal lesions with lower Map load showed a similar ordered granuloma pattern to the multifocal group, they had fewer than the other groups, though slightly higher than controls.

Map infection and macrophage epithelialization must be perceived as dynamic processes; epithelioid transformation is not immediate upon infection, and may arise from infection, sustained Map exposure, and/or chronic inflammation, not necessarily due only to Map infection. This consideration is particularly relevant when interpreting EM numbers and the barrier-like organization observed in animals with multifocal lesions, which should be taken with caution. The low Map load and reduced number of EMs in animals with focal lesions may be due to limited Map exposure and a rapid, efficient innate immune response. In contrast, animals with multifocal lesions likely experienced prolonged exposure due to the failure of the innate response containment. This failure led to the need for increased containment using alternative strategies, which could be related to a specific arrangement of EMs and the formation of mature granulomas. Thus, the number and organization of EMs may influence granuloma function and Map control. Previous studies investigated the severity of Map-associated granulomatous lesions and their relationship with clinical signs, bacterial load, fecal shedding, and immune responses, showing the dynamic and complex process between Map infection and granuloma formation (47, 48).

Various hypotheses about the consequences of epithelioid transformation of macrophages have been postulated; some propose that it engenders highly phagocytic and microbicidal cells (49), while others suggest that it produces non-phagocytic, secretory cells that enhance immune responses (50, 51). A longitudinal study (52) of granulomas induced by chronic *M. marinum* infection in frogs revealed that both epithelioid cell types (phagocytic and microbicidal cells/ non-phagocytic cells with secretory functions) could coexist in chronic granulomas, showing that even long-term lesions (long subclinical stage) are dynamic environments where bacterial replication and their phagocytic clearance maintain relatively stable bacterial numbers. Although granulomas tend to become more epithelioid over time, activated non-EMs persist alongside EMs. In our study, both non-EMs and EMs were found in DJE and DJELN, showing similar numbers in DJE and higher non-EM numbers in DJELN. Tissue-resident macrophages are extremely heterogeneous and tissue-specific (53). The differences observed between DJE and DJELN could be due to their distinct functions. DJE’s fundamental function is tissue homeostasis involving macrophages that participate in tissue repair and resolution of inflammation, whereas DJELN plays a central role in immunosurveillance. This functional heterogeneity may influence epithelioid differentiation processes. In agreement with this, we found more single-Iba1-positive phagocytic-like macrophages in DJELN and more tissue repair-like EMs in the DJE. Moreover, gut macrophages in DJE have a half-life estimated at 3 weeks (54), which means a continuous renewal, which may affect EMs accumulation.

The role of the granuloma in mycobacterial infections has been previously revised (55). Historically, it has been regarded as a host-protective structure that walls off the pathogen, constituting a barrier to bacterial proliferation and dissemination. Recent evidence suggests it is a dynamic structure that also promotes bacterial proliferation (56–58). It has been shown that mycobacterial granuloma formation in a Zebrafish-*M. marinum* model is accompanied by macrophage induction of canonical epithelial markers such as E-cadherin (21). They demonstrated that disruption of E-cadherin resulted in disordered granuloma formation, suggesting that the granuloma has a bacteria-protective role. They suggested that E-cadherin-expressing macrophages could be acting as an access barrier to protect bacteria from the host immune system in a similar structural disposition to that observed in the present study in animals with multifocal lesions. However, in our study (Table 2), animals presenting this EM pattern in the granuloma were subclinical and had a lower load of Map, indicating that the barrier seems to be controlling infection. In leprosy, a robust immune response results in paucibacillary disease, where well-developed granulomas contain scant organisms, whereas in multibacillary leprosy with poor granuloma formation shows uncontrolled bacterial growth. However, even paucibacillary leprosy can cause morbidity, indicating that an overexuberant granulomatous response may also harm the host (59). Curiously, animals with multibacillary leprosy, not able to control mycobacteria replication, present down-regulation of the expression of keratinocyte-associated genes (KRT14 and KRT5) (60). In tuberculoid leprosy, epithelioid giant cells and the formation of granulomas help restrict *Mycobacterium leprae*, whereas in lepromatous lesions, phagocytic foamy macrophages predominate and fail to control replication (61). Together, these findings suggest granuloma architecture may critically influence mycobacterial control across diseases.

Jenvey et al. (17) quantified macrophages in frozen bovine mid-ileum tissue sections from non-infected healthy cows, subclinically and clinically infected cows (mean ages 7-, 7-, and 5.7-year old, respectively). These authors reported a significant increase in macrophages with disease progression, even though many were not associated with Map. This suggested that while more macrophages are recruited to the site of infection, they fail to clear Map, which is transmitted from macrophage to macrophage, driving infection and the development of clinical disease. In a follow-up study, Jenvey et al. (18) characterized macrophage phenotypes in naturally Map-infected cattle. Clinical affected cows had significantly higher numbers of M2-like resolution/repair macrophages and fewer M1-like host defense macrophages, while subclinical cows had a balanced ratio and non-infected animals had more M1-like cells. In our study, the total number of macrophages (single Iba1 and double Iba1/CK) in DJELN and DJE was lower in non-infected controls than in infected animals; however, no significant differences were found between histopathological groups. Differences from Jenvey et al. (17) may reflect classification criteria (lesion type vs. disease stage), tissue type (fresh DJE/DJELN vs. frozen mid-ileum), technique (immunohistochemistry vs. immunofluorescence), primary antibody used [anti-Iba1 vs. anti-macrophage surface antigen (clone AM-3-K)], and quantification approach (granulomas only vs. total tissue section). Additionally, it cannot be ruled out that in the present study, other cells of the monocyte/macrophage lineage were counted using the anti-Iba1 antibody employed (42).

During maturation, several additional cell types are recruited into the granuloma that can define the impact and function of the granuloma during Map infection and disease progression. In this sense, we have observed the presence of single-CK-positive cells (light blue) in the granulomas that showed similar morphology and localization to RCs. Here, higher numbers of single-CK expressing cells were observed in DJELN and DJE granulomas of animals with multifocal lesions. Previous studies in humans showed the presence and distribution of CK-immunoreactive RCs in normal and pathological human lymph nodes (62, 63). RCs are normally present in lymphoid organs such as lymph nodes and play a crucial role in immune response to infection or inflammation (64). Moreover, RCs can constrain an excessive immune response (65). RCs were shown to be involved in transplantation tolerance, cancer immunity, and they are considered a key factor in autoimmune diseases (66, 67), which have been associated with Map infection. In this sense, RCs could also help to control Map infection; however, this is beyond the scope of the current study.

Overall, continuous exposure of animals to Map results in different outcomes within the herd, inside a dynamic balance where infection never gets established or is controlled by an efficient innate immune response in an important part of the farm population while in other individuals infection progresses to subclinical delimited focal or multifocal forms and in a smaller fraction of the herd to diffuse lymphocytic (cellular or Th1 type) or non-lymphocytic (humoral immune response or Th2 type) forms that will result in clinical disease (68). Cellular immunity may exert some early control, but responses appear to be very different among individuals, with different consequences on shedding and the development of a humoral response (69, 70). One factor in this variability may be the activation stage and organization of macrophages within granulomas. It is possible that an increase in the number of EMs located around the granuloma, forming an epithelioid barrier, might inhibit granuloma growth by disrupting the recruitment of newly activated macrophages to the structure, hindering mycobacterial macrophage-to-macrophage spread and thus serving to reduce their intracellular niche, favoring equilibrium towards EMs and walling off of the bacteria inside the granuloma. Although the transformation of macrophages into EMs is a dynamic and multifactorial process, the specific distribution of EMs, particularly in multifocal lesions, appears to be associated with resilience to PTB through the formation of ordered granulomas that limit tissue damage and bacterial dissemination. Replication in larger cohorts will be necessary to confirm our hypothesis and extend these observations.

## 5 Conclusion

The distribution pattern of EMs in the granulomas may influence the control of Map infection. Animals with multifocal lesions could control infection through the formation of granulomas, where EMs expressing CK, localized around the granuloma, form a structural and functional barrier that may avoid Map spread between macrophages and might limit or repair tissue damage, thereby protecting against clinical disease. Our results agree with the findings of Canive et al. (29), where a significant enrichment of the KRT pathway was observed in peripheral blood samples, at the gene expression level, in animals with multifocal lesions. Taken together, these findings raise the possibility that KRT genetic variants predispose cattle to develop multifocal lesions that maintain tissue resilience through the formation of organized granulomas that limit tissue damage and conthol the shift from subclinical to clinical disease. Whether this control is long-lasting needs to be further investigated, since there might exist external factors that trigger the loss of this control at the granuloma level, and therefore the progression of the disease. While our results support a potential role of cytokeratin expression in resilience to PTB, the relatively small sample size limits the generalizability of these findings. The number and, more importantly, the potential use of CK expression and EM distribution as resilience biomarkers should be regarded as a working hypothesis requiring further validation. These preliminary results need to be confirmed in larger studies, including more balanced subgroup sizes to strengthen the statistical power and to fully assess their relevance for PTB resilience.

## Data Availability

The raw data supporting the conclusions of this article will be made available by the authors, without undue reservation.
