# Supplementary figures and images for: Cytokeratin expression and distribution pattern of epithelioid macrophages in animals with different pathological forms of bovine paratuberculosis: potential role in resilience to PTB
Source: Front Vet Sci. 2025 Dec 11;12:1690841. doi: 10.3389/fvets.2025.1690841 (PMC12739959; doi:10.3389/fvets.2025.1690841)

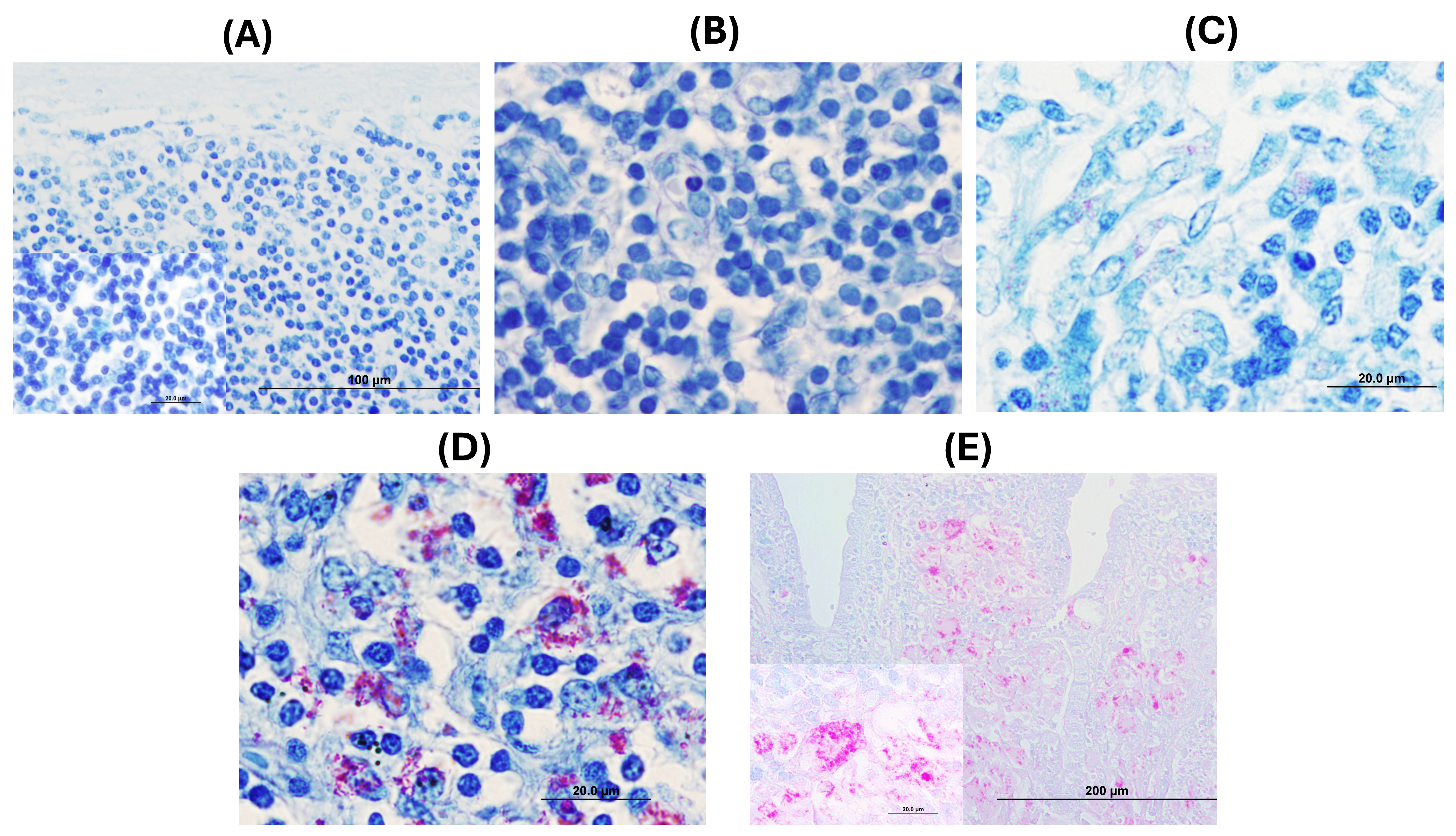

Supplement: Supplementary file 1 [file Image_1.TIF]

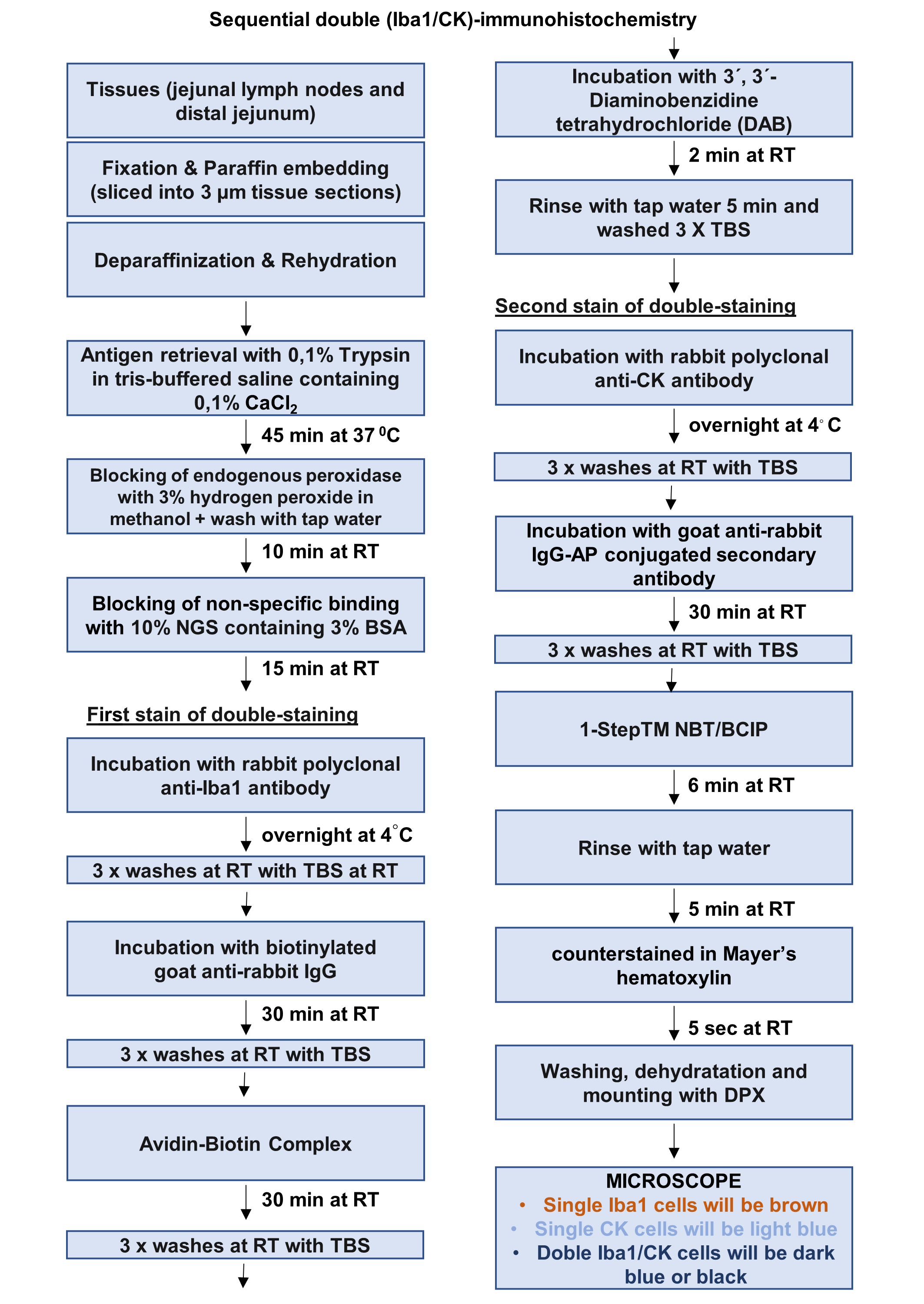

Supplement: Supplementary file 2 [file Image_2.TIF]

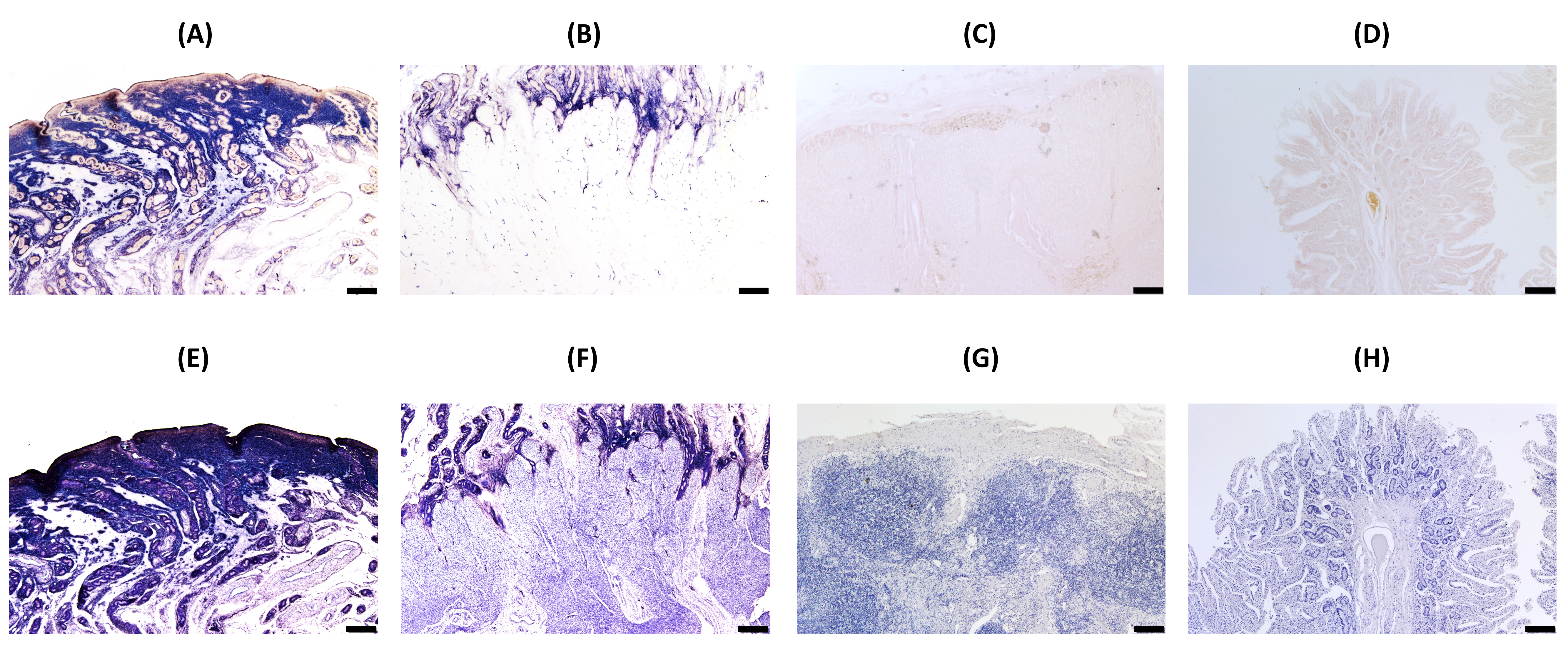

Supplement: Supplementary file 3 [file Image_3.TIF]

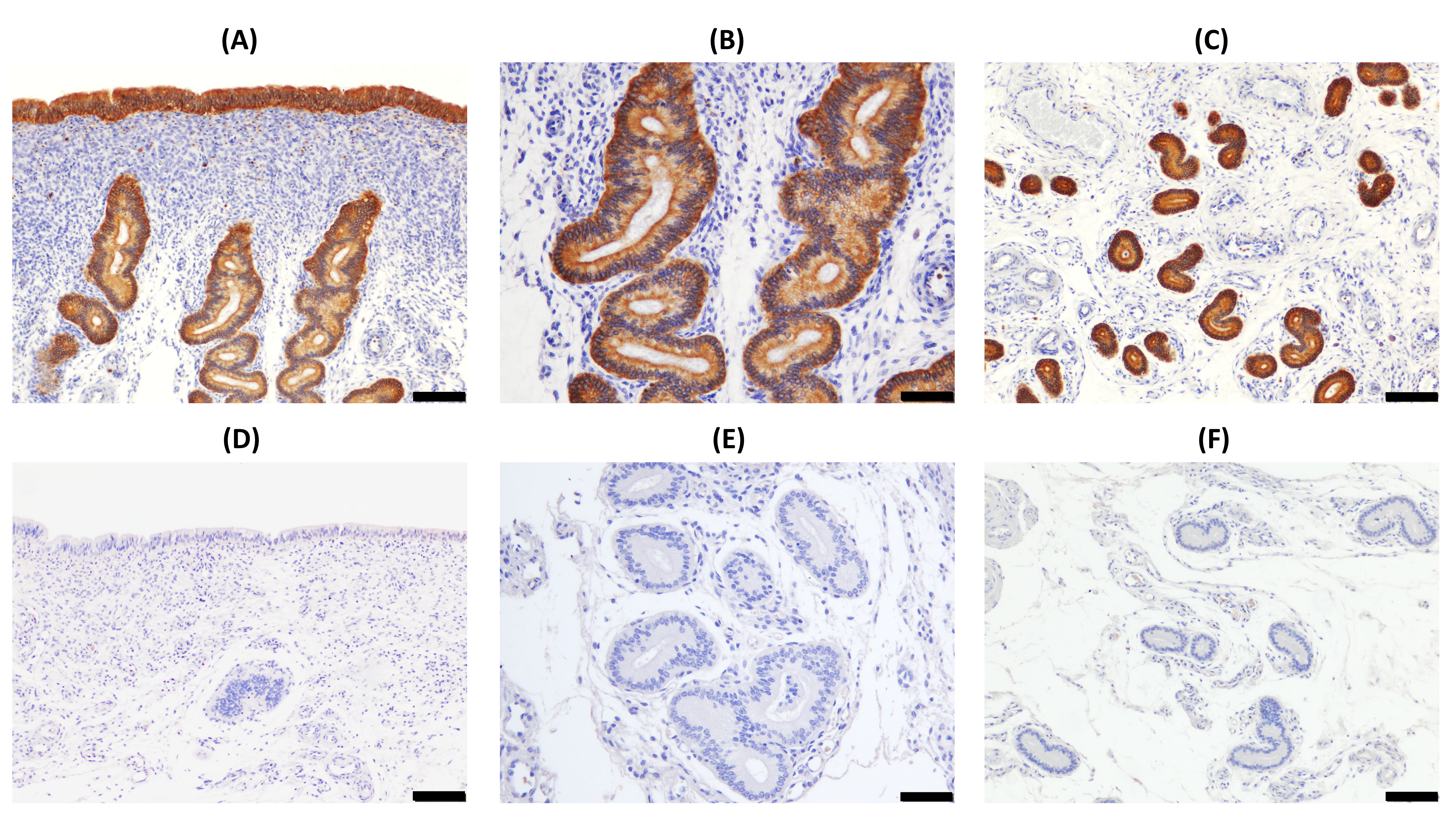

Supplement: Supplementary file 4 [file Image_4.TIF]

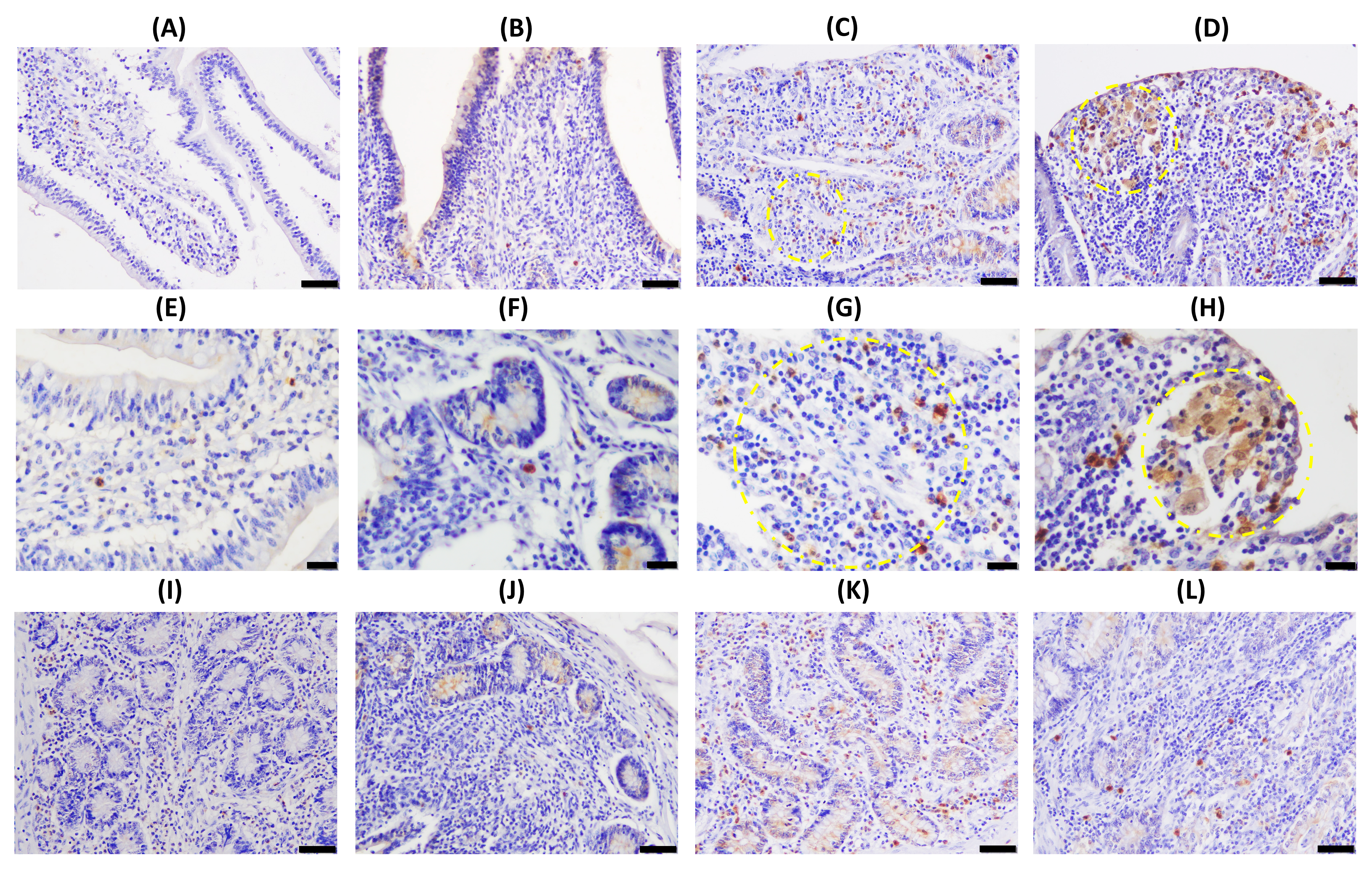

Supplement: Supplementary file 5 [file Image_5.TIF]

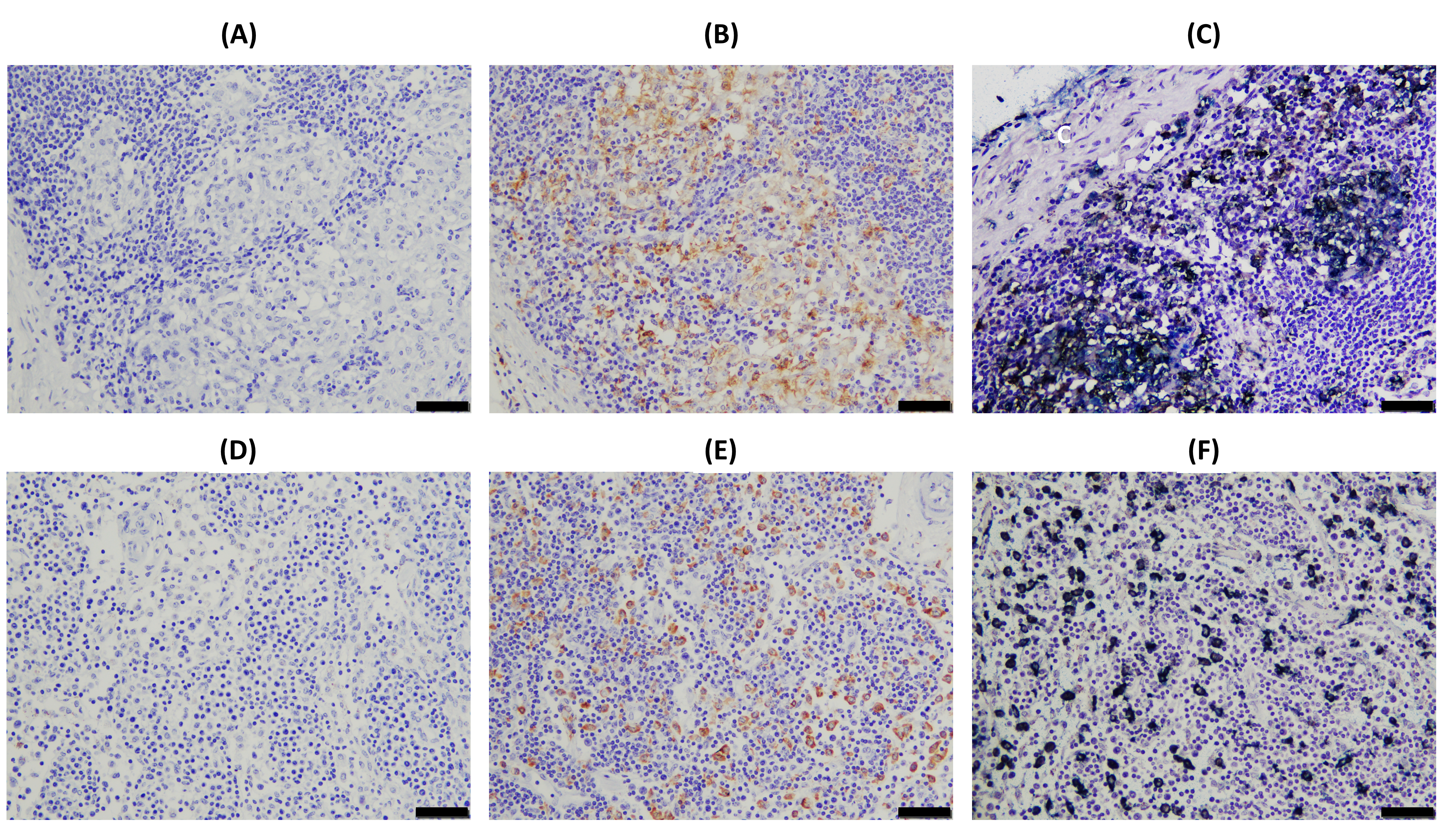

Supplement: Supplementary file 7 [file Image_7.tif]

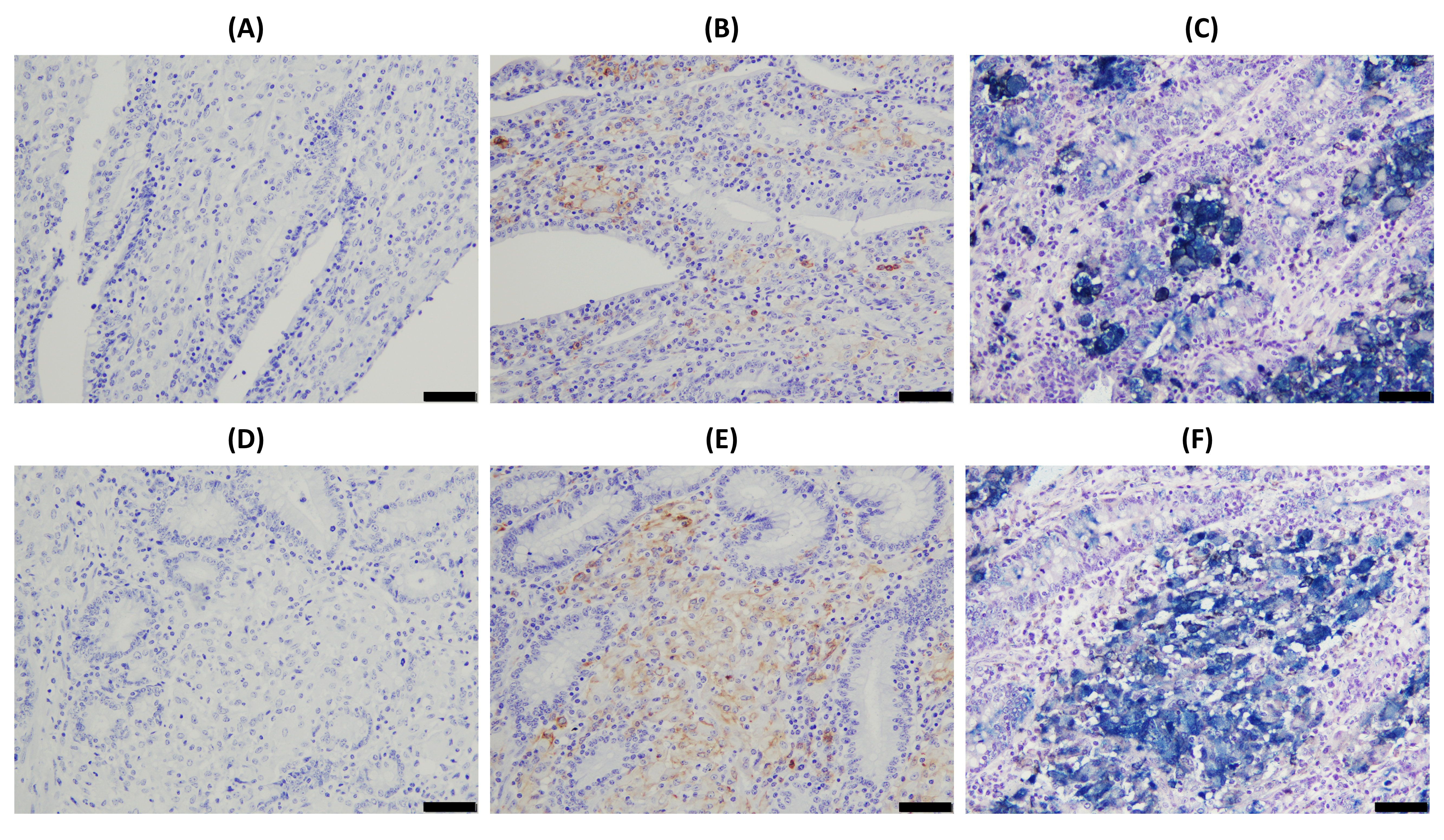

Supplement: Supplementary file 8 [file Image_8.tif]

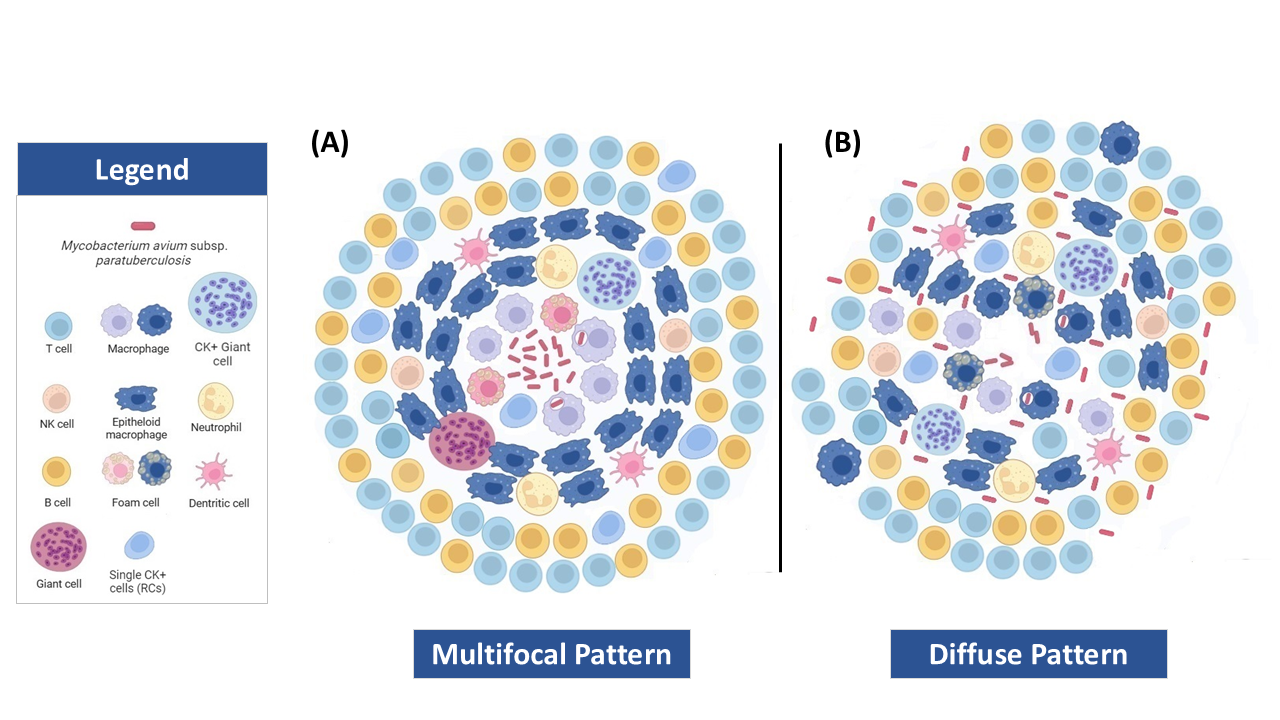

Supplement: Supplementary file 9 [file Image_9.tif]
